# *NOVA1* inhibition by miR-146b-5p in the remnant tissue microenvironment defines occult residual disease after gastric cancer removal

**DOI:** 10.18632/oncotarget.6542

**Published:** 2015-12-09

**Authors:** Sun Och Yoon, Eun Kyung Kim, Mira Lee, Woon Yong Jung, Hyunjoo Lee, Youngran Kang, You-Jin Jang, Soon Won Hong, Seung Ho Choi, Woo Ick Yang

**Affiliations:** ^1^ Department of Pathology, Yonsei University College of Medicine, Seoul, Korea; ^2^ Department of Pathology, Korea University Guro Hospital, Korea University College of Medicine, Seoul, Korea; ^3^ Department of Pathology, Kangbuk Samsung Hospital, Sungkyunkwan University College of Medicine, Seoul, Korea; ^4^ Department of Surgery, Korea University Guro Hospital, Korea University College of Medicine, Seoul, Korea; ^5^ Department of Surgery, Yonsei University College of Medicine, Seoul, Korea

**Keywords:** microenvironment, residual disease, hsa-microRNA-146b-5p, NOVA1, stromal cells

## Abstract

Occult residual disease in remnant tissues could be the cause of tumor relapse. To identify signal molecules and target cells that may be indicative of occult residual disease within a remnant microenvironment, proximal resection margin tissues of gastric cancers were used, as these correspond to the nearest remnant tissues after gastrectomy. Increased miR-146b-5p in the remnant microenvironment was determined to be a strong risk factor for tumor relapse and poor survival rate. *NOVA1*, a target gene of miR-146b-5p, was decreased in remnant tissues of patients with a poor prognosis. NOVA1 was enriched in stromal spindle cells such as fibroblasts within normal tissues. In non-neoplastic inflammation, such as gastritis, NOVA1 was highly enriched in T lymphocytes and stromal spindle cells, while expression of this protein was frequently decreased in those types of cells within gastric cancer tissues. Particularly, decreased NOVA1 in T cells within the gastric cancer tissues was correlated with decreased FOXP3-positive regulatory T cells and was associated with poor patient prognosis. *In vitro* analysis showed that the *NOVA1* gene was inhibited by miR-146b-5p. In immune cells as well as stromal spindle cells, decreased NOVA1, possibly inhibited by miR-146b-5p, is a candidate biomarker predicting poor prognosis of gastric cancer patients and is also a biomarker of occult residual disease in remnant tissues after gastric cancer removal.

## INTRODUCTION

Tumor recurrence is a major cause of cancer death. Occult residual disease may be the source of locoregional or metastatic recurrence after initial treatment. [1, 2] Therefore, early detection and control of occult residual disease is a critically important issue. Previous studies designed to detect occult residual disease prior to relapse have focused on detecting occult tumor cells. In studies of head and neck cancer, lung cancer, and prostate cancer, occult tumor cells not detected by conventional histologic examination were found in resection margin tissues using molecular methods involving DNA and RNA markers. The molecular markers detected in the resection margin tissues were correlated with recurrence in these studies.[3–6] Together with occult tumor cells, however, a tumor-promoting microenvironment and associated molecules could also be present in the remnant tissues of cancer patients, and these factors should also be considered when defining and controlling occult residual disease.

The microenvironment is an important contributor to the survival and growth of cancer cells. Tumor growth involves co-evolutionary processes among tumor cells, the extracellular matrix, the vasculatures, and immune cells.[7] The extracellular matrix, stromal spindle cells, vasculatures, and immune cells extend continuously from the original tumor field to the surrounding remnant tissues. Therefore, oncogenic changes that occur within the tumor microenvironment could extend into surrounding tissues and abnormally regulate resident cells in the remnant microenvironment. Although the remnant microenvironment may appear to be tumor cell-free in terms of conventional histology, ongoing molecular changes may result in clinically apparent tumor relapse. Therefore, investigation of occult residual disease should include consideration of the microenvironment and associated signaling molecules.

**Figure 1:**
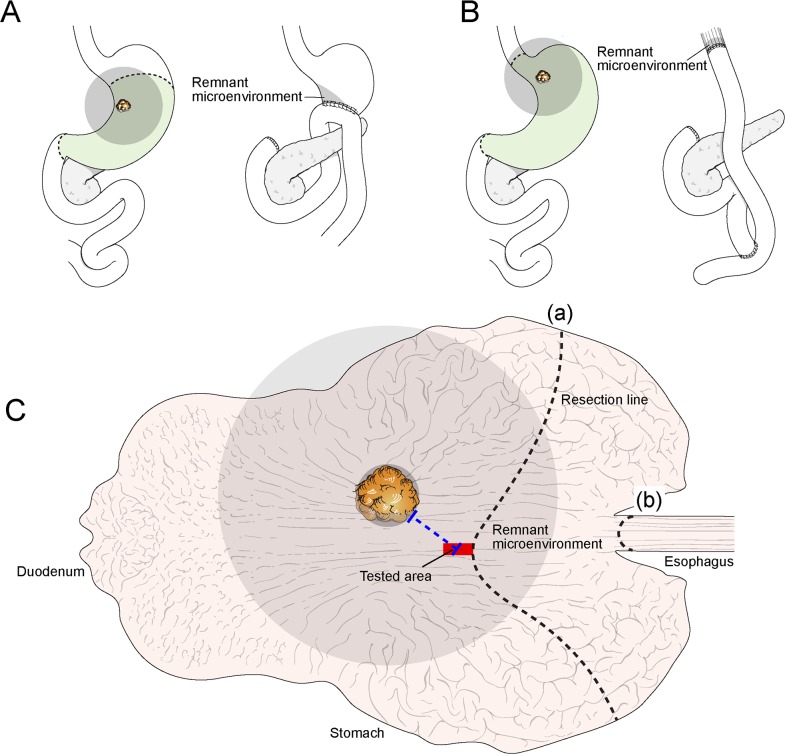
The sample model for studying remnant microenvironments Tumor cells, associated microenvironment cells, and signaling molecules could spread widely through the stromal matrix and vasculatures to the surrounding area (grey color). Part of the surrounding area (grey) influenced by the original tumor could remain even after removal of tumor and adjacent non-tumor tissues by subtotal gastrectomy **A.** or total gastrectomy **B.** Occult residual disease may be present in the remnant microenvironment (grey; A-C) after tumor removal. To prove occult residual disease in remnant tissues, the proximal resection margin section (tested area; red) was investigated; the proximal resection margin section was sampled from the resection line nearest from the original tumor mass in the case of subtotal gastrectomy (a) or total gastrectomy (b). Therefore, the proximal resection margin represents the section of remnant tissues nearest the original tumor mass. The microenvironment of the proximal resection margin area is located within the pathway where tumor-permissive or tumor-promoting changes extend into the nearest remnant tissues (grey); these changes could be the main component of occult residual disease.

In gastric cancer surgery, the tumor mass and adjacent normal tissues are radically resected, and the remnant tissues are anastomosed (Figure 1).[8] Therefore, resection-margin status is important, as tumor presence in this area infers that there may be residual tumor cells in the remnant tissues. A tumor-positive resection margin has been shown to have a significant negative effect on the survival of gastric cancer patients.[9–12] However, even after curative (R0) resection, which is defined as complete tumor resection with all margins histologically negative[9], and adjuvant systemic chemotherapy, locoregional and distant relapse frequently occur in advanced gastric cancer patients, resulting in treatment failure and cancer death.[13] Occult residual disease within the remnant tissues may be the cause of such recurrences in that the cellular and structural components of the microenvironment extend from the original tumor field to the remnant tissues.

In this study, we investigated whether we could detect occult residual disease in remnant tissue that was histologically tumor-free at the time of surgical cancer resection. Furthermore, we examined the effects of such residual disease on patient outcomes, such as recurrence and cancer death. In particular, we focused on epigenetic dysregulation that involved microRNA in order to detect occult residual disease in remnant tissues. We characterized abnormally regulated cells and the associated molecular changes within the microenvironment; these factors are the potential components of occult residual disease, which is correlated with a poor clinical outcome.

To detect occult residual disease in remnant tissues, we selected proximal resection margin tissue sections of gastric cancers. When considering conventional surgical and pathologic examination protocols for gastric cancer, the proximal resection margin tissue is defined as being located between the original tumor field and the remnant tissues. The site of the proximal resection margin is the counter section of remnant tissues nearest to the original tumor (Figure 1).[8, 14, 15] The microenvironment of the proximal resection margin area could be the shortest pathway by which potential occult residual disease crosses into remnant tissues; we hypothesized that this area was therefore the remnant microenvironment where occult residual disease, if present, would be detectable (Figure 1).

## RESULTS

### Profiles of aberrantly expressed miRNAs

Candidate microRNAs showing aberrant expression in the remnant microenvironment were identified using positive (*n* = 4) and negative (*n* = 4) controls of proximal resection margin tissues. The positive control samples were of proximal resection margin tissues from R1 resection containing low numbers of tumor cells and were obtained from gastric cancer patients who died of cancer relapse; the negative control samples were of tumor cell-free (R0) proximal resection margin tissues and were obtained from gastric cancer patients with a favorable prognosis (Supplementary Table S1). In microarray analysis containing probes specific for 1205 human and 144 human viral miRNAs, nine miRNA markers (hsa-miR-223-3p, -142-5p, -146b-5p, -150-5p, -363-5p, -532-5p, -502-3p, -1244, and -132-5p) were significantly increased and three (hsa-miR-933, -638, -3195) were significantly decreased in positive control samples compared to negative control samples. These 12 miRNAs were considered to be candidate miRNA markers. Expression data are summarized in Table 1 and Supplementary Data Set 1.

**Table:** The miRNA markers showing significant difference between positive controls and negative controls

miRNA	Chromosome locus	Log ratio	Fold change	Regulation	*P*-value
hsa-miR-223-3p	chrX	2.8	7.0	up	0.048
hsa-miR-142-5p	chr17	2.6	6.2	up	0.023
hsa-miR-146b-5p	chr10	2.3	5.0	up	0.007
hsa-miR-150-5p	chr19	2.3	4.9	up	0.032
hsa-miR-362-5p	chrX	1.0	2.0	up	0.003
hsa-miR-532-5p	chrX	0.8	1.7	up	0.012
hsa-miR-502-3p	chrX	0.7	1.7	up	0.038
hsa-miR-1244	chr2	0.7	1.6	up	0.048
hsa-miR-132-5p	chr17	0.6	1.5	up	0.041
hsa-miR-933	chr2	−0.8	−1.7	down	0.033
hsa-miR-638	chr19	−0.7	−1.6	down	0.004
hsa-miR-3195	chr20	−0.7	−1.6	down	0.009

The log ratio (log2 ratio) and the fold change are calculated by comparing the mean expression of each miRNA marker in positive controls and negative controls.

### miRNA markers correlated with poor oncologic outcomes

The 12 candidate miRNAs were analyzed in the set of proximal resection margin tissue samples (*n* = 140), which were determined to be histologically tumor-free (R0 resection) after curative radical surgery of gastric cancers. Changes in the expression of these candidate miRNAs were analyzed in terms of clinical and prognostic implications. Increased expressions of miR-146b-5p and miR-150-5p in the proximal resection margin tissues had significant clinicopathological implications and were correlated with poor oncologic outcomes. The overall correlations of the miRNA expression patterns of these two markers with clinicopathological factors and tumor relapse are summarized in Supplementary Table S2.

**Figure 2:**
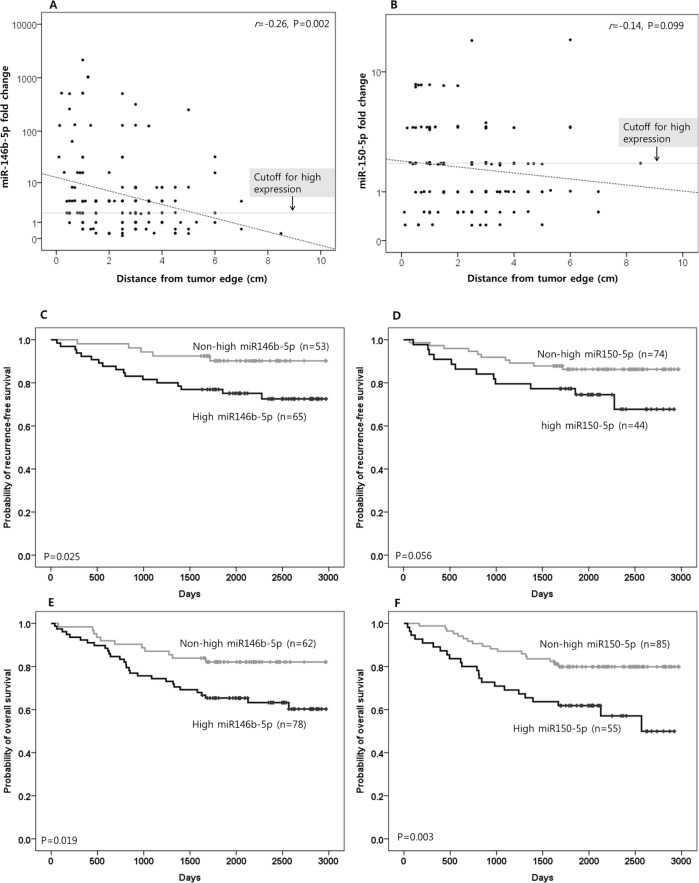
The clinical implications of miR-146b-5p and miR-150-5p The expression level of miR-146b-5p in the proximal resection margin section was negatively correlated with distance from the original tumor. However, high miR-146b-5p expression, which was defined as when the fold change was more than two fold the median value of normal gastric tissues of cancer-free individuals (cut-off for high expression), was also frequently observed in distant areas. **A.** For miR-150-5p, a similar tendency was noted, although with marginal significance **B.** The correlation coefficient (*r*) with statistical significance (P value) between the miR expression level and the distance from the original tumor was determined using the Pearson correlation test (A and B). On survival analysis for recurrence-free survival, high miR-146b-5p expression was associated with a shorter interval to recurrence/metastasis **C.** For miR-150-5p, a similar tendency was noted, though with marginal significance **D.** Information on the relapse (intraperitoneal recurrence and distant metastasis) was not clear in some cases due to lack of clinical, radiological, and/or pathological information; therefore, recurrence-free survival was analyzed in 118 cases that had reliable information for relapse during the follow-up time. In survival analysis for overall survival (OS), high miR-146b-5p and -150-5p expression were significantly correlated with inferior OS **E.** and **F.** (respectively). Survival function curves and survival rates for recurrence-free survival (C and D) and overall survival (E and F) were determined using the Kaplan-Meier method, and differences in survival rates were compared using the log-rank test.

miR-146b-5p expression in the tested microenvironment was negatively correlated with distance from the edge of the original tumor mass (*r* = −0.26, *P* = 0.002; Figure 2A). For miR-150-5p, a similar tendency was noted, though without statistical significance (*r* = −0.14, *P* = 0.099; Figure 2B). High expressions (fold change > two fold the median value of normal gastric tissues of cancer-free individuals) of miR-146b-5p and miR-150-5p were noted in 55.7% (78 of 140) and 39.3% (55 of 140) of tested cases, respectively. High expressions of miR-146b-5p and miR-150-5p were more frequently noted in higher pN-category tumors than in lower pN-category tumors (*P* = 0.050 and 0.008, respectively; Supplementary Table S2). There was no difference in miR-146b-5p or miR-150-5p expression according to the method of gastrectomy (subtotal distal gastrectomy versus total gastrectomy; Supplementary Table S2).

During the follow-up period after surgery and adjuvant therapy, recurrence within the intraperitoneal area, which corresponds to the locoregional area adjacent to the remnant microenvironment, occurred more frequently in cases with high miR146b-5p expression than in those with non-high miR-146b-5p expression (18.5% vs. 5.7%; *P* = 0.038). Cases with high miR-150-5p expression were also found to have more frequent intraperitoneal recurrence than those with non-high miR-150-5p expression, with the difference being marginally significant (20.5% vs. 8.1%; *P* = 0.052). High miR-146b-5p expression was more correlated with intraperitoneal recurrence and/or distant metastasis than non-high miR-146b-5p expression (26.2% vs. 9.4%; *P* = 0.020). For miR-150-5p, this tendency was noted with marginal significance (27.3% vs. 13.5%; *P* = 0.063). The results are summarized in Supplementary Table S2.

Based on the Kaplan-Meier survival analysis with a log-rank test for relapse-free survival, high miR-146b-5p expression was more correlated with a shorter interval to relapse (recurrence and/or metastasis) than non-high miR-146b-5p expression (*P* = 0.025; Figure 2C). For miR-150-5p, such a tendency was noted with marginal significance (*P* = 0.056; Figure 2D). Using the same method of analysis for overall survival (OS), high miR-146b-5p and miR-150-5p expression were found to significantly correlate with inferior OS (*P* = 0.019 and 0.003, respectively; Figure 2E and 2F). Based on a multivariate analysis using the Cox proportional hazards model, miR-146b-5p high expression was found to be an independent risk factor for inferior OS (Supplementary Table S3). The distance (safety margin) to the proximal or distal resection margin had no significant effect on survival.

### Potential target genes of miR-146b-5p

**Figure 3:**
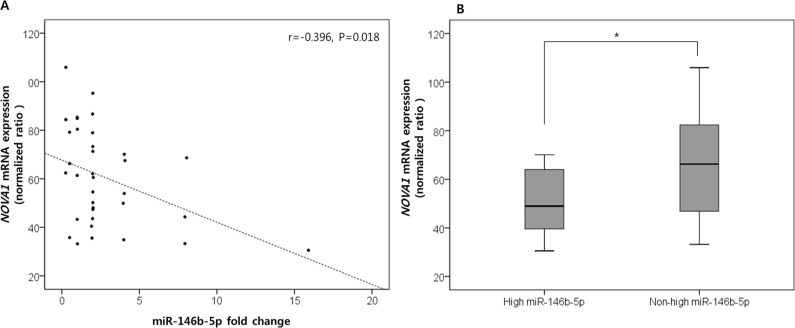
NOVA1 (neuro-oncological ventral antigen 1) as the predicted target genes of miR-146b-5p Among the predicted target genes of miR-146b-5p, *NOVA1* (*neuro-oncological ventral antigen 1*) expression was negatively correlated with the expression level of miR-146b-5p. The correlation coefficient (*r*) with statistical significance (P value) between the *NOVA1* mRNA expression level and the miR-146b-5p expression level was determined using the Pearson correlation test **A.** *NOVA1* mRNA levels significantly decreased in proximal resection margin tissues characterized by high miR-146 expression and poor oncologic outcomes compared to those with non-high miR-146b-5p expression and favorable oncologic outcomes. The statistical significance was determined by the Mann-Whitney U test **B.** Statistically significant differences are indicated by *, which signifies *P* < 0.05.

Considering that high miR-146b-5p expression in the histologically tumor-free microenvironment of proximal resection margin tissues was correlated with a poor oncologic outcome, we searched for potential target genes of miR-146b-5p by comparing proximal resection margin tissues from cases with high miR-146b-5p expression and poor oncologic outcomes (died of cancer relapse, *n* = 12) with those from cases with non-high miR-146b-5p expression and a favorable prognosis (*n* = 23). These cases were randomly selected among the set of 140 proximal resection margin samples after quality assessment of extracted mRNA. Using the microarray analysis containing probes specific for 27 predicted target genes of miR-146b-5p, *NOVA1* (*neuro-oncological ventral antigen 1*) mRNA expression was identified as a candidate target gene. The *NOVA1* mRNA expression level was negatively correlated with the expression level of miR-146b-5p (*r* = −0.396, *P* = 0.018; Figure 3A) and significantly decreased in the group with high miR-146 expression and poor oncologic outcomes compared to the group with non-high miR-146b-5p expression and favorable oncologic outcomes (*P* = 0.034; Figure 3B). Overall expression data are summarized in Supplementary Table S4 and Supplementary Data Set 2.

### Cellular localization of NOVA1 protein expression in non-tumor gastric tissues and gastric cancer tissues

**Figure 4:**
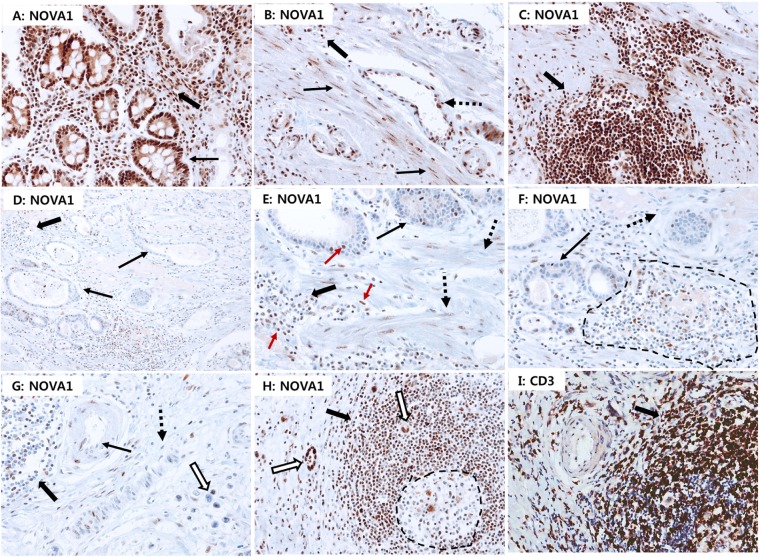

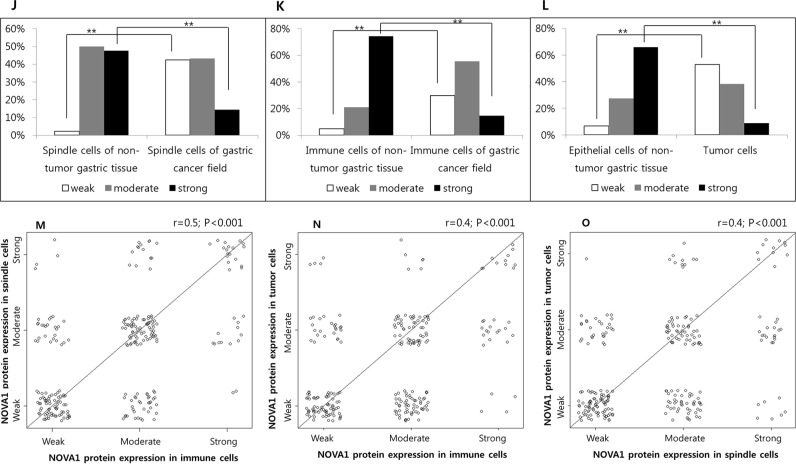
NOVA1 protein expression in non-tumor gastric tissues and gastric cancer tissues Strong NOVA1 expression was frequently observed throughout the microenvironment of non-tumor gastric tissues **A–C.** Epithelial cells of glands (thin arrow; A), stromal spindle cells such as fibroblasts or support cells (thin arrows; B), endothelial cells (dotted arrow; B) of the extracellular matrix, and immune cells of lymphoid structures (thick arrow; C) showed strong NOVA1 expression. Immune cells scattered within the lamina propria (thick arrow; A) and extracellular matrix (thick arrow; B) also showed strong NOVA1 expression. Immune cells expressing NOVA1 morphologically corresponded to lymphocytes (thick arrow; A-C). In the gastric cancer field **D–F.** negative or attenuated expression of NOVA1 was frequently observed in the overall microenvironment. Within the gastric cancer-embedded field, tumor cells (thin arrows; D-F), stromal spindle cells (dotted arrow; E-F), and immune cells of lymphoid structures (dotted area and thick arrows; D-F) frequently showed negative or attenuated expression of NOVA1. In another gastric cancer cases **G.** negative-to-attenuated NOVA1 expression was noted in immune cells (thick arrow), endothelial cells (thin arrow), fibroblasts or support cells (dotted arrow), and cancer cells (empty arrow). In the NOVA1-decreased cases (D-G), certain immune cells, stromal spindle cells, and epithelial cells of tumor glands occasionally showed positive NOVA1 expression, and such cells were admixed with many NOVA1-negative cells within the microenvironment (D-G; see the red-colored arrow in E). This feature may suggest the gradual attenuation of NOVA1 within the gastric cancer tissue field. In a gastric cancer case showing strong NOVA1 expression in the microenvironment **H.** most immune cells that expressed NOVA1 strongly (thick arrow; H) were identified as CD3-positive T lymphocytes (thick arrow); **I.** Most cells within the germinal centers, corresponding to the B cell zone, were negative for NOVA1 (dotted area; H). Tumor cells expressed NOVA1 strongly in this case (empty arrow; H). The microscopic figures were captured at x200 magnification power. When comparing the proportions of NOVA1 expression between non-tumor gastric tissues and gastric cancer tissues, weak NOVA1 expression in spindle cells (support cells, fibroblasts, endothelial cells) was more frequent, and strong NOVA1 expression was relatively less frequent in the gastric cancer field than in the non-tumor gastric tissue field **J.** For immune cells **K.** and epithelial cells **L.** similar results were observed. Statistically significant differences are indicated by **, which signify *P* < 0.001 as determined using the Chi-square test. The expression patterns of NOVA1 among immune cells and stromal spindle cells **M.** immune cells and tumor cells **N.** and stromal spindle cells and tumor cells **O.** were closely correlated to each other. The correlation coefficient (*r*) with statistical significance (P value) was determined using the Spearman correlation test.

In non-tumor gastric tissues (*n* = 45), strong NOVA1 protein expression was frequently observed in epithelial cells of gastric glands, immune cells of lymphoid structures, and stromal spindle cells, such as fibroblasts, reticular support cells, and endothelial cells, of the extracellular matrix spanning the gastric wall (Figure 4A–4C). In advanced gastric cancer tissues (*n* = 250), weak NOVA1 protein expression was frequently noted in tumor cells (epithelial cells) as well as in stromal spindle cells and immune cells within the original tumor mass-embedded microenvironment (Figures 4D–4G). Strong NOVA1 expression in the gastric cancer microenvironment was noted occasionally (Figure 4H and 4I).

When comparing the NOVA1 expression patterns in stromal spindle cells, immune cells, and epithelial cells (gastric gland cells or cancer cells) between non-tumor gastric tissues and gastric cancer tissues, the proportions of weak, moderate, and strong NOVA1 expression were different. In spindle cells (reticular support cells, fibroblasts, endothelial cells) of the gastric cancer field, weak NOVA1 expression was more frequent, and strong NOVA1 expression was relatively less frequent than cells of non-tumor gastric tissue (*P* < 0.001; Figures 4J). For immune cells and cancer cells (epithelial cells), similar expression patterns of NOVA1 were observed between gastric cancer tissues and non-tumor gastric tissues (both *P* < 0.001; Figure 4K and 4L). The expression patterns of NOVA1 among stromal spindle cells, immune cells, and tumor cells were closely correlated to each other (all *P* < 0.001; Figure 4M–4O).

**Figure 5:**
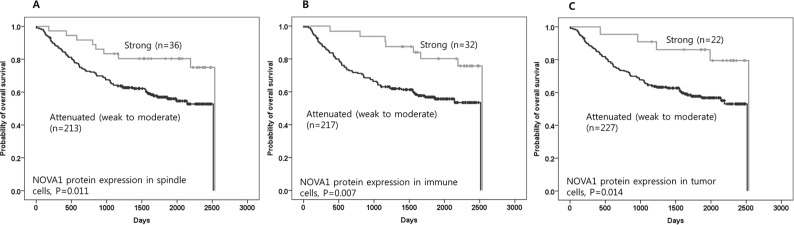
The prognostic meaning of attenuated NOVA1 expression in gastric cancer tissues Attenuated NOVA1 protein expression in spindle cells **A.** immune cells **B.** and tumor cells **C.** in gastric cancer fields was associated with inferior overall survival when compared to strong NOVA1 protein expression. The survival data was not available in one case, and analysis was performed using cases (*n* = 249) that had reliable information during the follow-up time. Survival function curves and survival rates were determined using the Kaplan-Meier method, and differences in survival rates were compared using the log-rank test.

In this set of advanced gastric cancer tissues, attenuated or suppressed NOVA1 protein expressions in stromal spindle cells, immune cells, and tumor cells were associated with inferior overall survival compared to cases with strong NOVA1 protein expression (*P* = 0.011, 0.007, and 0.014, respectively; Figure 5A–5C).

### Cellular localization of NOVA1 protein expression in the normal tissue microenvironment

Stromal spindle cells as well as immune cells are the major components of inflammation within the tissue microenvironment.[16] Inflammation or a tertiary lymphoid structure corresponds to ectopic lymphoid accumulations in response to stimuli, and the structure is made by various immune cells and matrix components.[16–20] The non-tumor gastric tissues we examined frequently had inflammation in the form of chronic gastritis or *Helicobacter* gastritis. Therefore, the expression pattern of NOVA1 that we observed in non-tumor gastritis may have reflected active inflammatory processes rather than the normal non-activated (inflamed) tissue condition. To identify NOVA1 protein expression in normal physiologic lymphoid structures (or secondary lymphoid structures), non-inflamed palatine tonsils were analyzed, as tonsils normally contain well-developed lymphoid structures.[19, 20]

**Figure 6:**
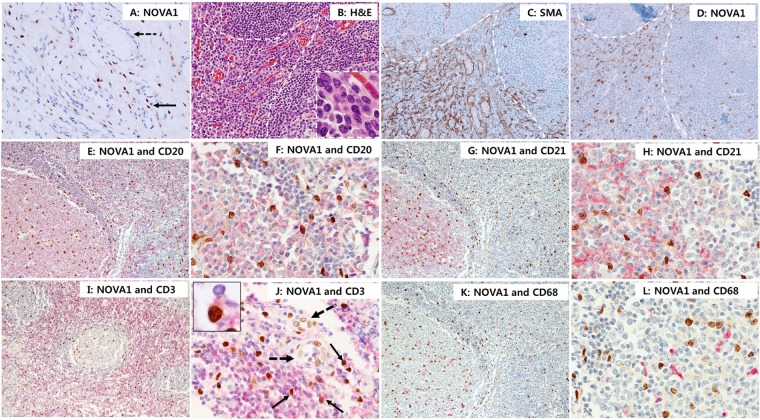
NOVA 1 expression in immune cells of lymphoid structures and stromal cells of the extracellular matrix In normal palatine tonsils, NOVA1-expressing cells had large, ovoid to spindle-shaped nuclei. These cells were frequently found as single isolated cells throughout the cortical zones of lymphoid follicles, the paracortical zones, and extracellular matrix (nuclear expression in brown; A-L). Stromal spindle cells (fibroblasts and support cells, thin arrow; A) throughout the extracellular matrix consistently expressed NOVA1. Endothelial cells in the vasculatures (dotted arrow; A) were negative for NOVA1 in the non-inflamed tonsil stroma **A.** In areas where alpha smooth muscle actin-positive myofibroblasts or fibroblasts were proliferating (white dotted area, see details of cell morphology within the high magnification inset; **B.** H&E staining; **C.** alpha smooth muscle actin staining), strong NOVA1 expression was localized **D.** Double staining with NOVA1 (nuclear expression in brown) and CD20 (cytoplasm/membrane expression in red); **E. and F.** and double staining with NOVA1 and CD21 **G. and H.** revealed that almost all B cells and follicular dendritic cells were not NOVA1-positive. In the paracortical T zone, most T cells were not NOVA1-positive based on double staining with NOVA1 and CD3 **I. and J.** A small number of T cells, however, were positive for both NOVA1 and CD3 (thin arrow and inset; J). In this case, endothelial cells (dotted arrow; J) expressed NOVA1 variably with weak to moderate intensity. Almost all CD68-positive macrophages and dendritic cells were negative for NOVA1 **K. and L.** The microscopic figures were captured at x100 and x200 magnification power.

In normal palatine tonsils (*n* = 10), cells with strong NOVA1 protein expression were distributed throughout the microenvironment (the cortical zone of lymphoid follicles, the paracortical zone, and the extracellular matrix). NOVA1-expressing cells were regularly scattered as single isolated cells (Figures 6A–6L). Scattered NOVA1-expressing cells had cytological features of fibroblasts or reticular support cells, with large-sized and ovoid-to-spindle-shaped nuclei. Stromal reticular support cells and fibroblasts in the extracellular matrix consistently showed strong NOVA1 expression (Figure 6A). In areas where alpha smooth muscle actin-positive stromal support cells, myofibroblasts, or fibroblasts were proliferating, NOVA1 was strongly enriched (Figures 6B–6D). S100 or c-Kit-positive spindle cells were also occasionally noted in the NOVA1-enriched area (Supplementary Figure S1). Mature endothelial cells expressed NOVA1 with variable intensity (from negative to moderate staining; Figures 6A and 6J). In the cortical and paracortical areas where immune B cells, T cells, and macrophages/dendritic cells were resident, almost all B cells, follicular dendritic cells, and macrophages/dendritic cells were negative for NOVA1. Although most mature small T cells were also negative for NOVA1, a small number of T cells did express this protein (Figures 6E–6L).

### NOVA1 protein expression in T cell and B cell lymphoma tissues

Given that only small numbers of T lymphocytes in normal palatine tonsils expressed NOVA1 among immune cells while most lymphocytes involved in inflammation observed in the gastritis cases expressed high levels of NOVA1, we concluded that NOVA1 is increased in activated T cells.

**Figure 7:**
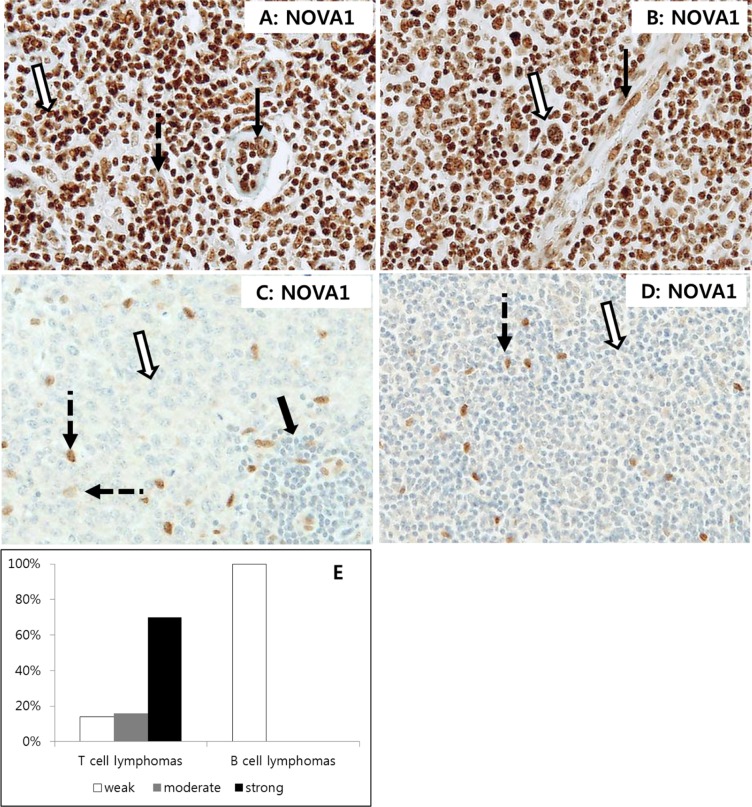
NOVA1 expression in T cell and B cell lymphomas In T cell lymphomas **A. and B.** strong NOVA1 expression was observed in tumor cells (empty arrow), fibroblasts (dotted arrow in A), and endothelial cells (black arrow). Staining results for angioimmunoblastic T cell lymphoma (A) and anaplastic large cell lymphoma (B) are shown. In B cell lymphomas **C. and D.** tumor cells were negative for NOVA1 (empty arrow). Cases showing NOVA1-expressing fibroblasts and support cells are presented (dotted arrow) to compare expression patterns among cells. Note that small mature immune cells of reactive lymphoid structures within the diffuse large B cell lymphoma field were negative for NOVA1 (thick arrow, C). A diffuse large B cell lymphoma **C.** and mantle cell lymphoma (D) are shown. Among the tested T cell lymphomas (*n* = 30), 70% showed strong NOVA1 expression, while all of the tested B cell lymphomas (*n* = 30) revealed weak (negative) NOVA1 expression (Figure E). The microscopic figures were captured at x200 magnification power.

To test this hypothesis, we evaluated NOVA1 expression in peripheral (mature) T cell lymphoma tissues (*n* = 30), which are characterized by high levels of T cell activation. In most cases of T cell lymphoma, tumor T cells as well as stromal fibroblasts and endothelial cells also frequently showed strong NOVA1 expression (Figures 7A–7B). To analyze NOVA1 expression in activated B cells, another type of immune cell, B cell lymphoma tissues (*n* = 30) were also investigated. All tumor B cells were negative for NOVA1 protein. Stromal spindle cells also frequently showed attenuated NOVA1 expression in most B cell lymphoma cases. Non-tumor immune cells involved in inflammation within the B cell lymphoma microenvironment were also frequently negative for NOVA1, although this observation was based on a small number of cases (Figures 7C–7D). Overall, 70% of tested T cell lymphomas (21 of 30) showed strong NOVA1 expression while none of the tested B cell lymphomas revealed strong NOVA1 expression (Figure 7E).

### Correlation of NOVA1 expression in immune cells with T cell density and regulatory T cell function

NOVA1 was increased mainly in activated T cells of non-tumor inflammation and T cell lymphomas. In immune cells of the gastric cancer field, however, NOVA1 expression was frequently decreased despite the histomorphologic features of inflammation formed in the gastric cancer field showing similarities with those of non-tumor inflammation, as in gastritis.

**Figure 8:**
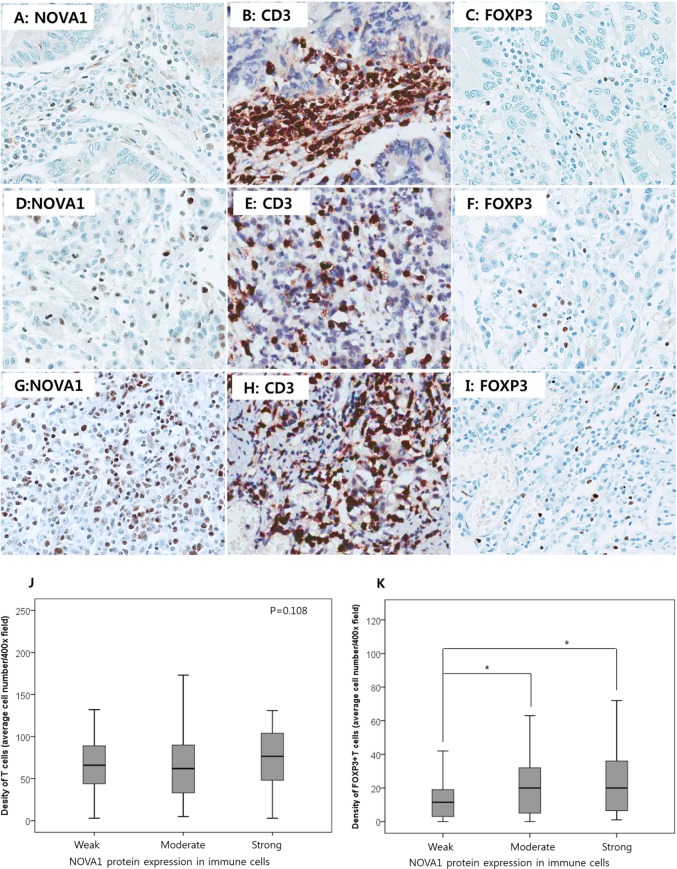
T cell density and regulatory T cell function according to NOVA1 expression in gastric cancer tissues In a case of weak NOVA1 expression in the immune cells **A.** many CD3-positive T cells are infiltrating into the gastric cancer field **B.** but FOXP3-positive regulatory T cells are rarely noted **C.** In a case of moderate NOVA1 expression in the immune cells **D.** some of the infiltrating CD3-positive T cells are positive for FOXP3 **E. and F.** In a case of strong NOVA1 expression **G.** CD3-positive T cells are admixed with the poorly differentiated gastric cancer cells **H.** and FOXP3-positive regulatory T cells are frequently noted **I.** The microscopic figures were captured at x400 magnification power. T cell density was not different but similar among cases showing weak (the case number, *n* = 97), moderate (*n* = 121), and strong (*n* = 32) NOVA1 expression in immune cells (T cells) of the gastric cancer tissue field **J.** However, the cell density of FOXP3-positive regulatory T cells decreased more in cases showing weak NOVA1 expression in immune cells than in cases of moderate or strong NOVA1 expression **K.** Densities (cell number) of CD3-positive T cells and FOXP3-positive T cells within the tumor microenvironment were counted in the five most representative 400-magnification high-power fields and then averaged after immunohistochemical staining with CD3 and FOXP3 in the set of gastric cancer tissues (*n* = 250). Statistically significant differences are indicated by *, which signifies *P* < 0.05 as determined using the one-way ANOVA test.

To determine whether decreased NOVA1 expression of immune cells (T cells) in the gastric cancer microenvironment is correlated with T exhaustion or abnormal function of regulatory T cells (Tregs), the density of T cells and FOXP3-positive T cells were counted using the set of gastric cancer tissues (*n* = 250; Figures 8A–8I). The T cell density was not significantly different according to NOVA1 expression in the immune cells; T cell density was similar among cases showing weak, moderate, and strong NOVA1 expression (Figure 8J). However, the cell number of Foxp3-positive T cells decreased more in cases showing weak NOVA1 expression than cases showing moderate or strong NOVA1 expression (Figures 8K) in the immune cells.

### NOVA1 protein expression in proximal resection margin tissues

**Figure 9:**
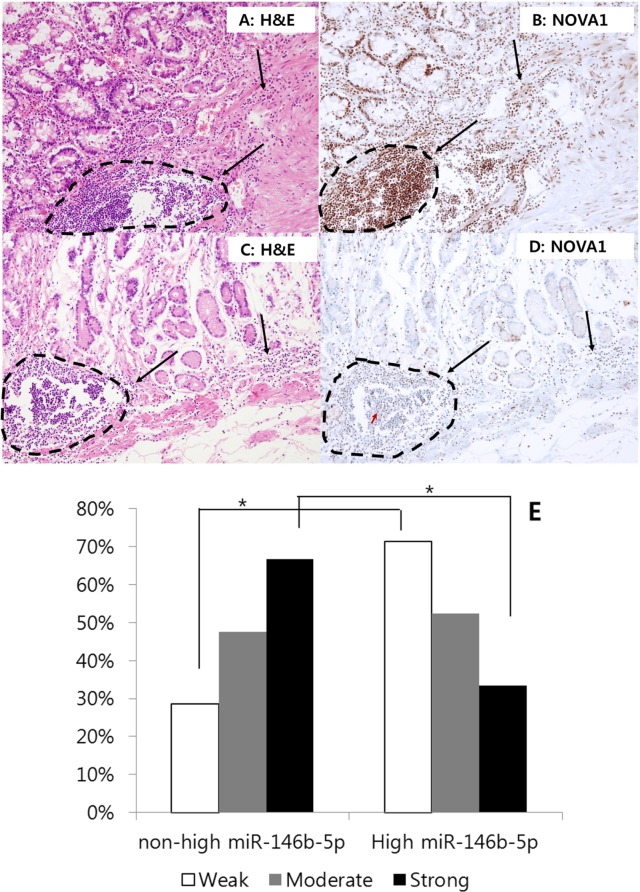
NOVA1 expression in the proximal resection margin tissues In the case of the non-high miR146b-5p and favorable prognosis group, strong NOVA1 expression was noted in immune cells of the proximal resection margin tissue **A. and B.** In the case of the high-miR146b-5p and poor prognosis group, weak NOVA1 expression was observed in immune cells of the microenvironment **C. and D.** The lymphoid structures of these cases appeared to be histologically similar; however, the NOVA1 expression level in the immune cells differed between these cases (dotted area and black arrow; A-D). In case (D) certain immune cells occasionally showed positive NOVA1 expression, and such cells were admixed with many NOVA1-negative cells within the microenvironment (see the red-colored arrow in D). This feature may suggest the gradual attenuation of NOVA1 within the microenvironment. The microscopic figures were captured at x200 magnification power. When comparing the proportions of NOVA1 expression in immune cells between the two groups (non-high miR146b-5p and favorable prognosis group versus high-miR146b-5p and poor prognosis group), weak NOVA1 expression was relatively more frequent, and strong NOVA1 expression was less frequent **E.** Statistically significant differences are indicated by *, which signifies *P* < 0.05 as determined using the Chi-square-test.

Given that NOVA1 was strongly expressed in non-tumor gastritis tissues while it was significantly attenuated in the gastric cancer field, we analyzed NOVA1 expression in histologically tumor cell-free proximal resection margin tissues. Among the set of 140 proximal resection margin samples, cases were randomly selected from those with high miR146b-5p expression and a poor prognosis (*n* = 34) as well as those with non-high miR-146b-5p expression and a favorable prognosis (*n* = 34). In the high-miR146b-5p and poor-prognosis group, the weak NOVA1 expression was relatively more frequent, and strong NOVA1 expression was relatively less frequent in immune cells of the microenvironment (Figures 9A–9E), although a statistically significant difference in NOVA1 expression was not observed for stromal spindle cells and epithelial cells.

### Inhibition of NOVA1 by miR-146b-5p in gastric cancer cells and fibroblasts

Endogenous NOVA1 expression level was higher in NHDF (fibroblast cell line) than in other tested cells: HUVEC (endothelial cell line) and SNU16 (gastric cancer cell line) cells expressed NOVA1 at low levels. NOVA1 gene expression was not detected in SNU5 (gastric cancer cell line) or SUN620 (gastric cancer cell line) cells (Supplementary Figure S2).

**Figure 10:**
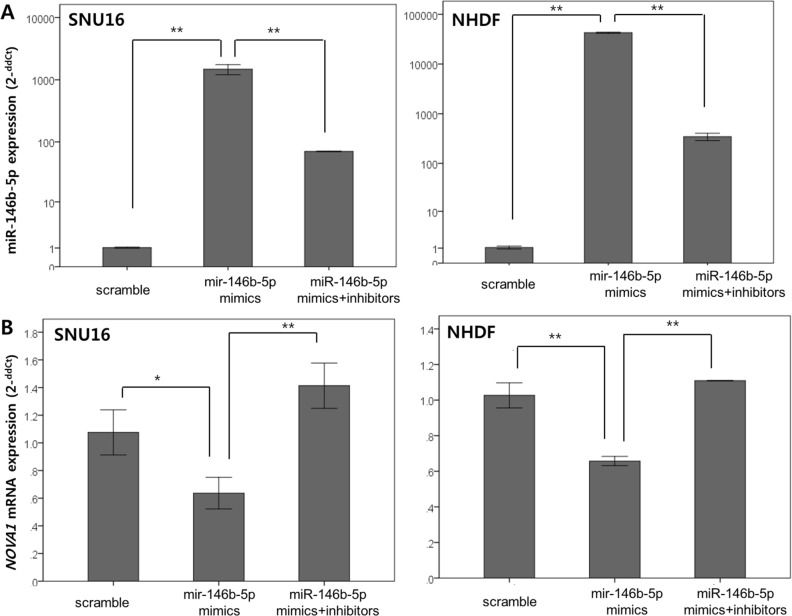

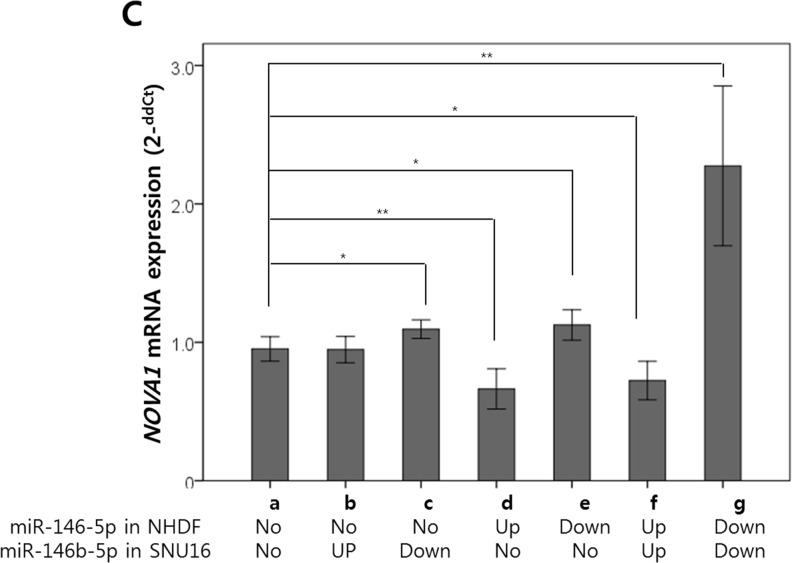
In vitro analysis of NOVA1 modulation by miR-146b-5p in gastric cancer cells, endothelial cells, and fibroblasts miR-146b mimics were transfected to increase miR-146b-5p expression, and both miR-146b mimics and miR-146b-5p inhibitors were co-transfected to decrease miR-146b-5p expression in SNU16 (gastric cancer cell line) and NHDF (fibroblast cell line) cells. At 72 hours after transfection, we confirmed that the level of miR-146b-5p increased or decreased according to the transfection **A.** According to the miR-146b-5p expression level **A.** variation of *NOVA1* mRNA expression was observed in NHDF and SNU16 cells **B.** The expression levels of miR-146b-5p and *NOVA1* mRNA were evaluated using quantitative reverse transcription PCR. Relative expression levels (2^−ddCt^) of miR-146b-5p and *NOVA1* mRNA were determined by comparing them with the respective levels of cells transfected with scrambled oligonucleotide in each cell line. The values shown in the histogram are means ± SD. Statistically significant differences are indicated by *, which signifies *P* < 0.05, and **, which signifies *P* < 0.001 as determined using two-sample *t*-tests (A and B). After the miR-146b mimics were transfected to increase miR-146b-5p expression (Up) and both miR-146b mimics and miR-146b-5p inhibitors were co-transfected to decrease miR-146b-5p expression (Down) in each SNU16 (gastric cancer cell line) and NHDF (fibroblast cell line) cell (upper lane), SNU16 and NHDF were co-cultured. At 72 hours after transfection, *NOVA1* mRNA expression was measured using quantitative RT-PCR. The *NOVA1* mRNA level of NHDF was analyzed and subsequently found to reciprocally decrease or increase in response to increased or decreased miR-146b-5p in NHDF cells themselves **C; a, d-g.** The NOVA1 mRNA level increased in response to transfected miR-146b-5p inhibitors of the co-cultured SNU16, even when NHDF cells were not transfected (C; a, c). The values shown in the histogram are means ± SD. Statistically significant differences are indicated by *, which signifies *P* < 0.05, and **, which signifies *P* < 0.005 as determined using the two-sample *t*-test. Relative expression levels (2^−ddCt^) of *NOVA1* mRNA were evaluated using quantitative reverse transcription PCR by comparing them with the respective levels of cells transfected with scrambled oligonucleotide or non-transfected cells. No, no transfection; Up, transfection of hsa-miR-146b-5p mimics; Down, transfection of both hsa-miR-146b-5p mimics and inhibitors.

After we increased or decreased miR-146b-5p expression through transfection with miR-146b-mimics and through co-transfection with both miR-146b mimics and miR-146b-5p inhibitors (Figure 10A), expression of the *NOVA1* mRNA level was measured in SNU16 cells (gastric cancer cell line) and NHDF cells (fibroblast cell line) using the quantitative reverse transcription polymerase chain reaction (RT-PCR) method (Figure 10B). At 72 hours after transfection, it was observed that expression of *NOVA1* mRNA was reciprocally decreased or increased in SNU16 and NHDF cells in response to miR-146b-5p expression (Figures 10A–10B).

To analyze possible changes of *NOVA1* in stromal cells through interaction with cancer cells according to miR-146b-5p expression, we used the co-culture system. After miR-146b mimics were transfected to increase miR-146b-5p expression and both miR-146b mimics and miR-146b-5p inhibitors were co-transfected to decrease miR-146b-5p expression in each SNU16 (gastric cancer cell line) and NHDF (fibroblast cell line) cell, SNU16 and NHDF cells were co-cultured. At 72 hours after transfection, *NOVA1* mRNA expression of NHDF was measured using quantitative RT-PCR. *NOVA1* mRNA reciprocally decreased or increased in response to increased or decreased miR-146b-5p in NHDF cells themselves (Figure 10C; a, d–g). Even when NHDF cells were not transfected, the *NOVA1* mRNA level reciprocally increased in response to transfected miR-146b-5p inhibitors of the co-cultured SNU16 cells (Figure 10C; a, c).

Cell survival, proliferation, and invasiveness did not change significantly during 72 hours of observation, nor did cell death (apoptosis or necrosis; Supplementary Figure S3).

## DISCUSSION

We investigated the characteristics of occult residual disease in remnant tissues, as this is related to poor patient prognosis. We hypothesized that if tumor-promoting changes remain in the remnant tissues, the remnant microenvironment may be the reservoir for tumor relapse. Considering that the microenvironment is an organized complex of epithelial cells, the structural matrix, fibroblasts, endothelial cells, and immune cells, we investigated the predominant molecular changes and associated cell types in the remnant microenvironment that were thought to be components of occult residual disease related to poor clinical outcomes. We selected proximal resection margin tissue sections of gastric cancers, as the proximal resection margin represents the nearest remnant microenvironment where occult residual disease is likely to be present. The microenvironment of the proximal resection margin area is located within the pathway where potential occult residual disease could spread into the nearest remnant tissues (Figure 1).

Several miRNAs (hsa-miR-223-3p, -142-5p, -146b-5p, -150-5p, -363-5p, -532-5p, -502-3p, -1244, -132-5p, -933, -638, and -3195) were aberrantly expressed in proximal resection margin tissues which contained tumor cells at the microscopic level from patients who underwent R1 resection and died of cancer recurrence. These markers were considered to be the predominant epigenetic markers of the remnant microenvironment containing low numbers of tumor cells that survived after being separated from the original mass and then progressed into clinically apparent tumor relapse.

Several studies have demonstrated that microRNAs are present in resident cells within the tumor microenvironment[21]; however, most miRNAs found in the present study have not previously been reported in earlier studies. Such previous studies used cell lines or a single cell type microdissected from the microenvironment in which the tumor mas was embedded. Differences in study models may therefore account for this discrepancy between studies.

The 12 miRNAs were validated in terms of their clinical implications using a set of advanced gastric cancer cases that had a tumor-free proximal resection margin (R0). Among these, increased miR-146b-5p was significantly correlated with cancer relapse and poor survival outcomes. Increased miR-146b-5p in the proximal resection margin tissues was correlated with intraperitoneal (locoregional) recurrence and distant metastasis even after completing curative treatment. In addition, increased miR-146b-5p was correlated with an inferior overall survival rate and determined to be an independent prognostic factor. These findings suggest that increased miR-146b-5p in the remnant microenvironment may be a strong predictor of tumor recurrence and poor patient outcome. Only a small number of studies on miR-146b-5p have been published. Recent reports have demonstrated the role of miR-146b-5p in cancer growth; miR-146b-5p is involved in the epithelial mesenchymal transition (EMT) through regulation of PRRX1 or ZNRF, which are EMT markers, [22, 23] and it is also involved in nuclear factor kB (NF-kB) signaling via regulation of TRAF6 during cancer development and immune reaction [24].

The present study was not about miR-146b-5p function on cancer cells. We focused on the remnant tissues in which the microenvironment was histologically tumor cell-free yet the patient had a poor prognostic outcome. In the present study, the *NOVA1* gene was identified as the target gene inhibited by miR-146b-5p in the tumor cell-free proximal resection margin tissues characterized by poor clinical outcomes. *NOVA1* (neuro-oncological ventral antigen 1) is known to be involved in the neuronal splicing program [25–28]; the role of *NOVA1* has been rarely investigated in other types of cells or cancers.

To find target cells correlated with *NOVA1* dysregulation, we performed a morphoproteomic analysis using immunohistochemistry. This method is one of the best tools for cytological and histological localization and identification of cells expressing translated target genes within the complex tissue microenvironment. The changes of NOVA1 expression were distinctively different in immune cells and in stromal spindle cells, such as fibroblasts, reticular support cells, and endothelial cells, according to the tissue conditions as follows: normal physiologic lymphoid tissues, non-tumor gastric tissues, gastric cancer tissues, immune T or B cell malignancy tissues, and tumor-free proximal resection margin tissues with or without increased miR-146b-5p expression.

Among immune cells, NOVA1 expression was restricted to T lymphocytes. In the non-activated (inflamed), physiologic secondary lymphoid structures of the palatine tonsils, only small numbers of T cells expressed NOVA1. This NOVA1 expression increased in most T cells when involved in non-neoplastic inflammatory conditions, such as chronic gastritis or *Helicobacter* gastritis. Tumor T cells (T cell lymphomas) also demonstrated increased NOVA1 expression. In gastric cancer fields, however, NOVA1 protein expression was frequently decreased in immune cells (T cells) within the tumor microenvironment. In addition, decreased NOVA1 expression in immune cells in the gastric cancer field correlated with poor patient prognosis. The histomorphological features of inflammation (or tertiary lymphoid structures) [16–20] within the gastric cancer field were similar to those in non-tumor gastritis tissues. However, the immune surveillance provided by inflammation may have been dysregulated in the NOVA1-decreased gastric cancer field.

We noted that decreased NOVA1 expression in T cells was not the result of the depletion or exhaustion of T cells. However, cell numbers of FOXP3-positive T cells were more decreased in gastric cancer cases showing weak NOVA1 expression in T cells. Transcription factor forkhead box P3 (FOXP3) is known to be the master regulator in regulatory T cells; therefore, it is also known to be the most specific marker for the identification of regulatory T cells (Tregs). Tregs are well known to play a central role in the immune response to tumors as well as various disease conditions. Lack or paucity of FOXP3-positive Tregs is known to lead to immunodysregulation. [29, 30]. The decreased NOVA1 expression in immune cells (T cells) seems to be correlated with suppressed Tregs in the gastric cancer field. The suppressed Tregs may lead to immune dysfunction in the NOVA1-decreased gastric cancer field. A small number of recent studies show the presence of the FOXP3-miR-146a/b-NF-κB axis in breast cancer or prostate cancer, although such studies focus on cancer cells and direct binding between miR-146a and FOXP3.[31, 32] From these findings, however, it could be suggested that increased miR-146b-5p may be involved in the suppression of FOXP3-positive Tregs directly or indirectly through NOVA1 dysregulation or other signaling pathways.

Interestingly, T cell lymphomas, which are extreme examples of T cell activation, showed strong NOVA1 expression in both tumor cells and stromal cells. B cells of normal tonsils (normal, physiologic secondary lymphoid structures) and B cell lymphomas did not express NOVA1. Stromal spindle cells mixed with B cell lymphomas also frequently showed attenuated NOVA1 expression. Given that the NOVA1 expression level was closely correlated between immune cells, stromal spindle cells, and tumor cells as shown in gastric cancer tissues, changes of NOVA1 expression may be shared among resident cells of the microenvironment. In tumors with increased NOVA1 expression, such as T cell lymphomas, strong NOVA1 expression may be induced in stromal spindle cells or vice versa. In tumors with attenuated or negative NOVA1 expression such as B cell lymphomas, upstream signaling molecules such as miR-146b-5p may inhibit NOVA1 in overall resident cells within the microenvironment. In fact, several recent studies have revealed that changes of miRNAs in the tumor microenvironment likely occur by crosstalk among resident cells through various signaling methods such as autocrine and/or paracrine manners.[21].

In the present study, fibroblasts, stromal spindle cells, and/or reticular support cells of the extracellular matrix within the microenvironment constitutively express NOVA1 in normal or non-neoplastic inflammatory conditions. In fact, a recent study showed that *NOVA1* was enriched in normal fibroblasts independent of tissue or organ origin and suggested that NOVA1 may be involved in the splicing programs of normal fibroblasts. [33] The present study noted that NOVA1 expression was frequently decreased in stromal fibroblasts and spindle cells in the gastric cancer field. In addition, decreased NOVA1 expression in stromal fibroblasts and spindle cells of the gastric cancer field was related to poor patient prognosis.

Stromal spindle cells are major components of tissue inflammation or tertiary lymphoid structures within the microenvironment. [16–18] Recent studies showed that stromal spindle cells and fibroblasts actively regulate immune cells in the inflammatory process and are involved in immune escape of cancers.[16, 34, 35] In addition, fibroblasts and stromal spindle cells, or cancer-associated fibroblasts (CAFs), are major components of the tissue matrix.[21, 35, 36] The extracellular matrix has been shown to be involved in tumor cell growth, angiogenesis, invasion, and immune escape.[34, 35] Decreased NOVA1 function in stromal spindle cells may also be involved in the escape of aggressive cancers from immune surveillance. miR-146b-5p may be a regulator of fibroblasts through inhibiting *NOVA1* expression. If fact, CAFs are known to be epigenetically regulated through miRNAs.[21, 37] Considering that *NOVA1* is an alternative splicing factor, deregulated splicing programs inhibited by miR-146b-5p in fibroblasts and the stromal matrix may be associated with tumor progression in remnant tissues.

Given that the findings of *NOVA1* expression was closely correlated among resident cells of the microenvironment, we attempted to demonstrate the possible interaction among stromal cells and cancer cells in the process of *NOVA1* expression as a response to miR-146b-5p. In the study, the *NOVA1* mRNA level was increased in fibroblast cell lines in response to miR-146b-5p inhibitors, which were transfected to the co-cultured gastric cancer cells even when fibroblast cells themselves were not transfected by miR-146b-5p inhibitors. Although we didn't observe changes of *NOVA1* by possible crosstalk of miR-146b-mimics, it was suggested that crosstalk of molecules may occur between cells within the microenvironment. Our hypothesis could be also supported by several recent studies on the crosstalk of miRNA signals among resident cells [21].

As for the sources of increased miR-146b-5p in the tumor cell-free remnant tissues, secondary spreading through the stromal matrix or vasculatures from the original tumor site may be possible, as the expression of miR-146b-5p increased as the distance from the original tumor decreased. However, even in relatively distant areas from the original tumor site, increased miR-146b-5p was also frequently observed. Recent evidence indicates that primary changes could also occur in mesenchymal stroma, and alteration of resident cells in the microenvironment could initiate cancers. [38] Increased miR-146b-5p could be the primary change in resident cells within remnant tissues.

We made several new important findings, although several of these require additional confirmation in future studies. Particularly, our findings of decreased NOVA1 in the immune cells, stromal spindle cells, and cancer cells were more specific to gastric cancer tissues than to non-tumor gastric tissues. In addition, decreased NOVA1 in the gastric cancer field was associated with poor patient prognosis. Therefore, the NOVA1 expression pattern could be a candidate biomarker that could be easily tested during routine diagnosis using human samples and immunohistochemistry. The usefulness of NOVA1 immunostaining could be extended to various cancer types after further validation studies.

In summary, occult residual disease can remain in histologically tumor cell-free remnant tissues after removal of cancers, and this occult residual disease is a strong risk factor for tumor relapse. Specifically, increased miR-146b-5p in the proximal resection margin tissues, representing the nearest remnant tissues after gastrectomy, was significantly associated with tumor relapse and poor outcomes in gastric cancer patients. miR-146b-5p inhibited expression of *NOVA1*, an alternative splicing factor, in immune cells and stromal spindle cells within the remnant microenvironment. Immune cells and stromal spindle cells with abnormal NOVA1 expression could be the main component of occult residual disease in remnant tissues after tumor removal and are therefore potential causes of tumor relapse.

## METHODS

### Selection of candidate microRNAs

Candidate microRNAs that showed aberrant expression in the remnant microenvironment and were possibly related to aggressive oncologic outcomes were identified using clinical samples of the proximal resection margin tissues and microarray analysis. Positive control samples (*n* = 4) were obtained from R1 resection cases (microscopically tumor-present in the resection margin) [9, 15] with poor oncologic outcomes (died of cancer recurrence), and the volume of tumor cells was found to be less than 10% of the entire tested area. We reasoned that these controls were appropriate for investigating the potential molecular changes within the surrounding microenvironment, in which low numbers of tumor cells may have survived, interacting with resident cells of the remnant microenvironment. Negative control samples (*n* = 4) were obtained from cases of R0 resection (microscopically tumor-free resection margin) [9, 15] with a favorable clinical outcome (survived without any event during a follow-up time of more than 5 years). Detailed clinicopathological information on the control cases is provided in the supplementary information and Supplementary Table S1.

Total RNA for microarray experiments was extracted (GENOCHECK Ltd., Seoul, Korea) using two slices of formalin-fixed paraffin-embedded (FFPE) tissue sections dissected at a thickness of 10-mm. The quality and quantity of RNA were checked using an Agilent Bioanalyzer (Agilent Technologies, CA, USA) and determined to be appropriate for microarray expression analysis. Microarray analysis was performed using Agilent Human miRNA arrays (8x60K v16.0, Agilent Technologies), which contained probes specific for 1205 human and 144 human viral miRNAs. Candidate miRNAs were selected when the expression level of an individual miRNA differed significantly (P-value < 0.05) between the positive and negative controls and the fold change was >1.5 based on comparison of the mean values of the positive and negative controls (Supplementary Data Set 1).

### Clinical validation of selected candidate miRNAs

The expression pattern of selected candidate miRNAs in the microenvironment was analyzed in the set of proximal margin tissues obtained from 140 advanced gastric cancer cases. These cases had undergone radical surgery and curative R0 resection (tumor-negative in the proximal and distal resection margin lines).[9, 15] Standard gastrectomy, which included resection of at least two-thirds of the stomach and D2 lymph node dissection, was performed by gastric surgery specialists at Gangnam Severance Hospital between 2005 and 2008. No preoperative treatment was performed. The histopathology for each case was reviewed by pathologists. The median follow-up period was 67.4 months (range, 0.7 to 101.8 months). Clinicopathological details are described in the supplementary information and Supplementary Table S5. For negative controls, we used proximal resection margin tissue samples from 10 cancer-free individuals who had undergone gastrectomy at Gangnam Severance Hospital between 2005 and 2008 due to an ulcer or other benign inflammatory lesion.

FFPE tissues submitted as the proximal margin section were prepared under the conventional surgical and pathologic examination protocols (Figure 1).[8, 14, 15] The tested area was histologically confirmed to be tumor-free on microscopic observation. RNA, including microRNA, was isolated using the miRNeasy FFPE Kit (Qiagen, Hilden, Germany). cDNAs were synthesized using looped reverse transcription primers specific to each miRNA species. Looped reverse transcription and forward and reverse primers for each microRNA (hsa-miR-223-3p, -142-5p, -146b-5p, -150-5p, -363-5p, -532-5p, -502-3p, -1244, and -132-5p, as well as a housekeeping gene, U6sn) are listed in Supplementary Table S6. Expression of miRNA species was assayed quantitatively. In brief, a 20 μl mixture containing 2 μl of cDNA, 2 U of i-Taq DNA polymerase (iNtRON, Seongnam, Korea), 2 μl of 10X i-Taq reaction buffer (iNtRON), 2 μl of 2.0 mM dNTP mixture (Takara, Japan), 1 μl of 5 pmol/μl forward primer, 1 μl of 5 pmol/μl reverse primer, 0.9 μl of 5X SYBR Green (ROCHE, Switzerland), and 10.9 μl of tertiary distilled water was prepared, and amplification was performed using a StepOnePlus Real-Time PCR instrument (Applied Biosystems, CA, USA) under the following conditions: denaturation at 94°C for 5 min followed by 30 cycles of 94°C for 15 sec and 66°C for 40 sec (fluorescence signal acquisition was performed during this phase). Immediately after amplification, melting curve analysis was performed. All samples were analyzed twice to confirm reproducibility. Using StepOnePlus Real-Time PCR System software (version 2.1; Applied Biosystems), the expression value (2^−ddCCT^) of each case was normalized to the median expression value of negative controls. High expression was defined when the normalized value was >2 (fold-change > 2).

### Finding target genes of the miRNA

For the validated miRNA that had significant clinical implications, predicted target messenger RNAs were queried in a web-based database (http://mirecords.umn.edu/miRecords; http://www.microrna.org/microrna), and 27 highly relevant genes and three reference genes were selected to form a custom-based array set. FFPE tissues of the proximal resection margin section were selected from the above set of proximal resection margin tissues (*n* = 140). To create a positive control group, 24 cases with abnormal expression of the identified miRNA in the proximal resection margin tissues and a poor clinical outcome were selected randomly. For the negative controls, 24 stage-matched cases with no aberrant expression of the miRNA and a favorable prognosis were selected. After quality assessment of the extracted RNA, 12 cases from the positive control group and 23 cases from the negative control group were ultimately analyzed. Clinicopathological information on these control samples is summarized in Supplementary Table 7. Expression analysis was performed using nCounter Gene Expression Custom CodeSet (30 Genes × 48 Reactions; NanoString Technologies, Seattle, WA, USA). Predicted target gene expression is summarized in Supplementary Data Set 2.

### Clinical samples for cellular localization of translated target genes

We investigated the expression of the target protein of the candidate miRNA in each cell type making up the microenvironment, using FFPE tissues from clinical samples.

Normal palatine tonsil tissues (*n* = 10), a set of non-tumor gastric tissues (*n* = 45), and a set of advanced gastric cancer tissues (*n* = 250) were included in the analysis. Tonsil tissues were obtained via tonsillectomy from cancer-free individuals. A set of normal gastric tissue (*n* = 45) was used as a control, representing a non-tumor gastric microenvironment. A set of gastric cancer tissues (*n* = 250) was used to analyze the tumor-embedded microenvironment. These gastric cancer tissues were obtained from surgical resection performed at Korea University Guro Hospital from 2002 to 2005. Details regarding this set of gastric cancer tissues are summarized in the supplementary information and Supplementary Table 8.

For comparative analysis, mature (peripheral) T cell lymphoma tissues (*n* = 30) and mature B cell lymphoma tissues (*n* = 30) were included as examples of activated immune cells. The subtypes were as follows: peripheral T cell lymphoma (not otherwise specified, *n* = 15), angioimmunoblastic T cell lymphoma (*n* = 5), anaplastic large cell lymphoma (*n* = 10), diffuse large B cell lymphoma (*n* = 20), follicular lymphoma (*n* = 5), mantle cell lymphoma (*n* = 2), and marginal-zone B cell lymphoma (*n* = 3). All lymphoma tissue samples were reviewed by hematopathologists (SOY and WIY) based on current World Health Organization (WHO) criteria.[39]

Representative tissue sections of palatine tonsils and tissue microarrays for non-tumor gastric tissue, gastric cancer tissues, and lymphoma tissues were prepared.

### Immunohistochemistry

Immunohistochemistry was performed using a Ventana Bench Mark XT Autostainer (Ventana Medical Systems, Tucson, AZ, USA) and a LEICA BOND-III Autostainer (Leica Biosystems, Newcastle Upon Tyne, UK). Tested primary antibodies were as follows: NOVA1 (dilution 1:500; Abcam, Cambridge, UK), CD68 (dilution 1:150; DAKO), CD20 (dilution 1:1600; clone L26; DAKO), CD3 (dilution 1:200; LabVision, Fremont, CA, USA),

CD4 (dilution 1:200; Cell Marque, Rocklin, CA, USA), FOXP3 (dilution 1:100; Aviva Systems Biology, CA, USA), myeloperoxidase (dilution 1: 2000, DAKO), CD34 (dilution 1:50; clone QBEnd 10; DAKO), S100 (dilution 1:2000; DAKO), and alpha smooth muscle actin (dilution 1:500; clone 1A4; DAKO). Double staining with NOVA1/CD20, NOVA1/CD3, NOVA1/CD21, and NOVA1/CD68 was performed using palatine tonsil tissues for cellular localization of NOVA1 expression. Cell typing of immune cells and stromal spindle cells was also confirmed histomorphologically with H&E and immunohistochemical stains as follows: macrophages/monocytes/dendritic cells (CD68, S100, myeloperoxidase), B-lymphocytes (CD20), T-lymphocytes (CD3), regulatory T cells (FOXP3), neutrophils (myeloperoxidase), stromal spindle cells (Schwann cells, fibroblasts, support cells, and endothelial cells; S100, smooth muscle actin, and CD34).

### Grading of NOVA1 protein expression

Pathologists, including the hematopathologist, evaluated the cellular localization of NOVA1 expression for each cell type within the microenvironment under microscopic examination. NOVA1 expression scoring was semiquantitatively determined by staining intensity grade (1, no staining to weak intensity; 2, moderate intensity; 3, strong intensity) multiplied by the percentage grade of positive cell nuclei (1, 0%–9%; 2, 10%–19%; 3, 20%–29%; 4,30%–39%; 5, 40%–49%; 6, 50%–59%; 7, 60%–69%; 8, 70%–79%; 9, 80%–89%; 10, 90%–100%) after modifying the previously-used semiquantitative approach.[40] The resulting NOVA1 protein expression scores ranged from 1 to 30 and were classified as having strong (21-30), moderate (11-20), or weak (1-10) expression.

### Density of T cells and FOXP3 expression

Cell densities (number) of T cells and FOXP3-positive T cells were investigated according to NOVA1 expression in T cells. Using the set of gastric cancer tissues (*n* = 250), the densities of CD3-positive T cells and FOXP3+ T cells that had infiltrated the tumor microenvironment were counted as follows according to the method of our previous study:[41] the five most representative 400-magnification high-power fields were selected. Preserved intact nuclei were counted manually, and the counted cell numbers were averaged.

### Cell lines and reagents

Gastric cancer cell lines SNU-5 (00005; KCLB, Seoul, South Korea), SNU-16 (00016; KCLB) and SNU-620 (00620; KCLB) were purchased and maintained in RPMI1640 medium (22400-089, Gibco; Life Technologies, CA, USA) supplemented with 10% FBS (6000-044, Gibco; Life Technologies). Human umbilical vein endothelial cells (HUVEC; CC-2517; Lonza, Basel, Switzerland) were purchased and maintained in EGM (CC-3124; Lonza), and human dermal fibroblasts (NHDF; CC-2511;Lonza) were purchased and maintained in FGM-2 (CC-3132; Lonza).

### Transfection

Scrambled control miRs (scrambled oligonucleotides for miR-146b-5p and miR-146b-5p inhibitors; AccuTarget™ miRNA mimic Negative control; Bioneer, Daejeon, South Korea), a hsa-miR-146b-5p mimic (Bioneer), and a hsa-miR-146b-5p inhibitor (Bioneer) were obtained and transfected at a concentration of 30 nM using lipofectamine reagent (L3000008, Invitrogen; Life Technologies). After 72 hours, cells were harvested. Experiments were performed independently three or more times. An Annexin V/propidium iodide (PI) assay (FITC Annexin V Apoptosis Detection Kit; BD Pharmingen, CA, USA) was performed to determine cell apoptosis or necrosis after transfection.

### Co-culture of cell lines

SNU16 cells were co-cultured with NHDF cells on 6-well plate polycarbonate membrane transwell inserts (Costa3412; Corning, MA, USA). After transfection of scrambled control miRs, the hsa-miR-146b mimic, and both the hsa-miR-146b mimic and the hsa- miR-146b-5p inhibitor, cells were co-cultured. After 72 hours, cells were harvested. Experiments were performed independently three or more times.

### RNA isolation and quantification PCR

miRNA was isolated using a mirVana miRNA isolation kit (AM1560, Ambion; Life Technologies), and cDNA was constructed using the TaqMan MicroRNA Reverse Transcription kit (4366596, AB; Life Technologies) for miR146b-5p and U6. TaqMan probes used were as follows: has-mir-146b (001097, AB; Life Technologies) and U6 snRNA (001973, AB; Life Technologies). The average relative expression of miR146b-5p was determined by the comparative method (2^−ddCt^). After RNA isolation using the RNeasy Plus mini kit (74134; Qiagen, Hilden, Germany), cDNA was constructed using a cDNA synthesis kit (11754-050, Invitrogen). TaqMan probes used were as follows: *GAPDH* (Hs99999905-m1, AB; Life Technologies) and *NOVA1* (Hs00359592-m1, AB; Life Technologies). Quantitative PCR was performed using an ABI StepOnePlus™ (Applied Biosystems) with the following cycling parameters: 50°C for 2 min, 95°C for 5 min, and 40 cycles of 95°C for 15 sec and 60°C for 1 min. Relative mRNA expression levels of *NOVA1* were determined by the comparative method (2^−ddCt^).

### Statistics

The Mann-Whitney (MW) U test, one-way ANOVA, two-sample *t*-test, *x*^2^ test, and Pearson's or Spearman's correlation test were used to analyze the significance of differences among the variables examined. Overall survival times were measured from the date of surgery to the date of death or last follow-up visit. Recurrence-free survival was defined as the time from surgery to the first clinical, radiological, and/or histological evidence of recurrence in intraperitoneal or distant organs. Patient survival rates were determined using the Kaplan-Meier method, and differences in survival rates were compared using the log-rank test. Multivariate analysis was performed using the Cox proportional hazards model. A two-sided P-value <0.05 was considered statistically significant. Statistical analyses were performed using IBM SPSS 22 software for Windows (IBM Corp, Somers, New York).

### Study approval

The Institutional Review Board of Gangnam Severance Hospital, Yonsei University College of Medicine approved this study.

## SUPPLEMENTARY INFORMATION FOR METHOD SECTIONS






