# Supplementary material for: NOVA1 inhibition by miR-146b-5p in the remnant tissue microenvironment defines occult residual disease after gastric cancer removal
Source: Oncotarget. 2015 Dec 9;7(3):2475–95. doi: 10.18632/oncotarget.6542 (PMC4823049; doi:10.18632/oncotarget.6542)
Supplement: Supplementary file 1 [file oncotarget-07-2475-s001.pdf]

SUPPLEMENTARY INFORMATION FOR METHOD SECTIONS

Selecting candidate microRNAs that may be present in the tumor-associated microenvironment of aggressive gastric cancers

Four positive control patients died of gastric cancer progression after radical distal gastrectomy with adjuvant chemotherapy within 5 years of their initial diagnosis. Negative controls included four gastric cancer cases that underwent radical distal gastrectomy with adjuvant chemotherapy and were pathologically determined to be tumor-free in the test area. These four cases were age- and stage-matched to positive controls; negative control patients showed no cancer-related events such as recurrence or cancer-death during 5 or more years of follow-up and were still alive at the time of the present study. Surgical resections were performed at Gangnam Severance Hospital between 2005 and 2008. The proximal resection margin tissue section was sampled from the area where the distance from the tumor edge to the resection line was the shortest, under the conventional surgical and pathologic examination protocols [1–3] (Figure 1). Detailed information about the control cases is presented in supplementary Table 1. A 1.5 to 2 cm-length area of whole gastric wall tissue sections was included in the analysis.

Clinical validation of selected candidate miRNAs

Clinical validation of the selected candidate miRNAs was performed in 140 advanced gastric cancer cases. These cases had undergone radical surgery and curative R0 resection (tumor-negative in the proximal and distal resection margin line). Surgical resection was performed at Gangnam Severance Hospital (Seoul, Korea) between 2005 and 2008. Standard gastrectomy, which includes resection of at least two-thirds of stomach and D2 lymph node dissection, was performed by one specialist in gastric surgery. No preoperative treatment was performed. Among patients, 92.5% of indicated patients received 5-FU and/or platinum-based adjuvant chemotherapy; oral prodrug or the intravenous form was administered on a case-by-case basis.

FFPE tissues submitted as the proximal margin section were prepared under the conventional surgical and pathologic examination protocols (Figure 1). [1–3] To avoid bias caused by potential occult residual disease in the distal remnant area of the duodenum, we investigated only cases in which the distance to the proximal margin line was shorter than the distance to the distal margin line, considering that potential occult tumor cells, tumor-promoting microenvironment cells, and related molecular changes spread radially from the original mass field (Figure 1).

Clinico-pathological information was obtained, and resected tumors were staged according to the seventh AJCC/UICC (American Joint Committee on Cancer/International Union Against Cancer Classification) TNM cancer classification system. [4] World Health Organization and Japanese classifications were used for histologic tumor typing. [3] Based on the Japanese classification, differentiated type histology included well to moderately differentiated papillary and tubular adenocarcinoma, while undifferentiated type included poorly differentiated adenocarcinoma, signet-ring cell carcinoma, and mucinous adenocarcinoma. [3]

Cellular localization of translated target genes

For the advanced gastric cancer set of samples, tissue microarrays of gastric tissue samples were made with FFPE specimens obtained from surgical resection performed at Korea University Guro Hospital from 2002 to 2005. No preoperative treatment was performed. Standard gastrectomy, which includes resection of at least two-thirds of stomach and D2 lymph node dissection, was performed by one specialist in gastric surgery. Based on tumor stage, 91.6% of cases received postoperative 5-fluorouracil (FU)-based adjuvant chemotherapy. Tumors were classified according to the 7th edition of the American Joint Committee on Cancer (AJCC) tumor, node, metastasis classification system and the Japanese classification system. The World Health Organization and Japanese classification were used for histologic tumor typing.[3, 4] The mean follow-up period was 46.3 months. Clinicopathological characteristics are summarized in supplementary Table 8. All lymphoma tissue samples were reviewed by pathologists including reference hematopathologists (SOY and WIY) based on current World Health Organisation (WHO) criteria.[5]

REFERENCES

1. Japanese Gastric Cancer A. Japanese gastric cancer treatment guidelines 2010 (ver. 3). Gastric cancer : official journal of the International Gastric Cancer Association and the Japanese Gastric Cancer Association. 2011;14:113–23.
2. Rosai J, and Ackerman LV. Rosai and Ackerman's surgical pathology. New York: Edinburgh ;Mosby; 2011.
3. Japanese Gastric Cancer A. Japanese classification of gastric carcinoma: 3rd English edition. Gastric cancer : official journal of the International Gastric Cancer Association and the Japanese Gastric Cancer Association. 2011;14:101–12.
4. Edge SB, and American Joint Committee on Cancer. AJCC cancer staging manual. New York ; London: Springer; 2010.
5. Swerdlow SH, International Agency for Research on C, and World Health O. WHO classification of tumours of haematopoietic and lymphoid tissues. Lyon, France: International Agency for Research on Cancer; 2008.

SUPPLEMENTARY FIGURES AND TABLES

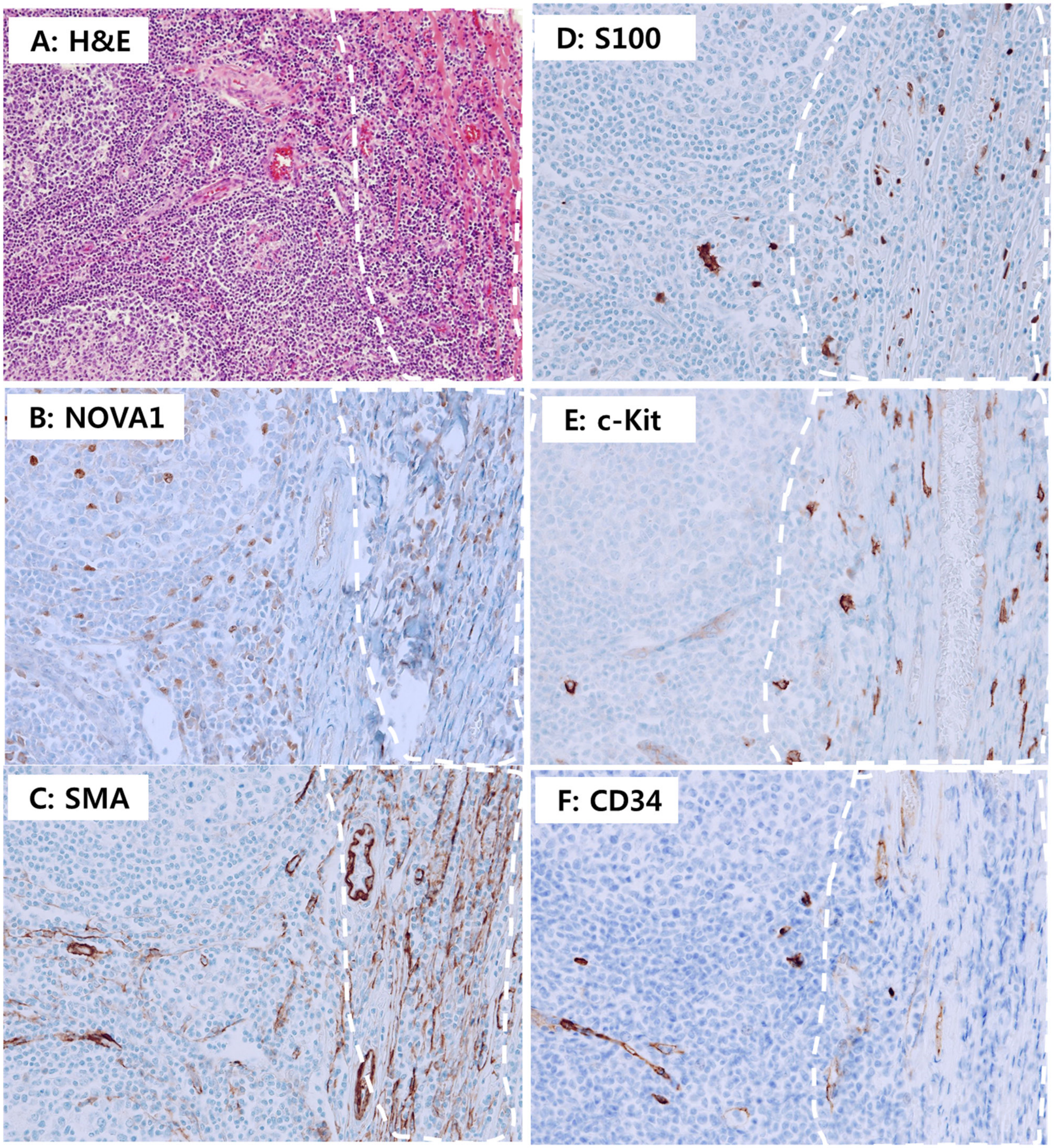

Supplementary Figure S1: NOVA1 expression in various stromal cells of extracellular matrix. In the area of collagenous fibrosis (dotted area); **A.** NOVA1-expressing cells were enriched **B.** In this area, alpha smooth muscle actin-positive spindle cells were proliferating, indicating that some cells were (myo) fibroblasts **C.** S100 or c-Kit positive spindle cells were also noted in the NOVA1-enriched area **D. and E.** (respectively). CD34-positive mature endothelial cells were rarely found in this area **F.** All figures were captured at X100 magnification power.

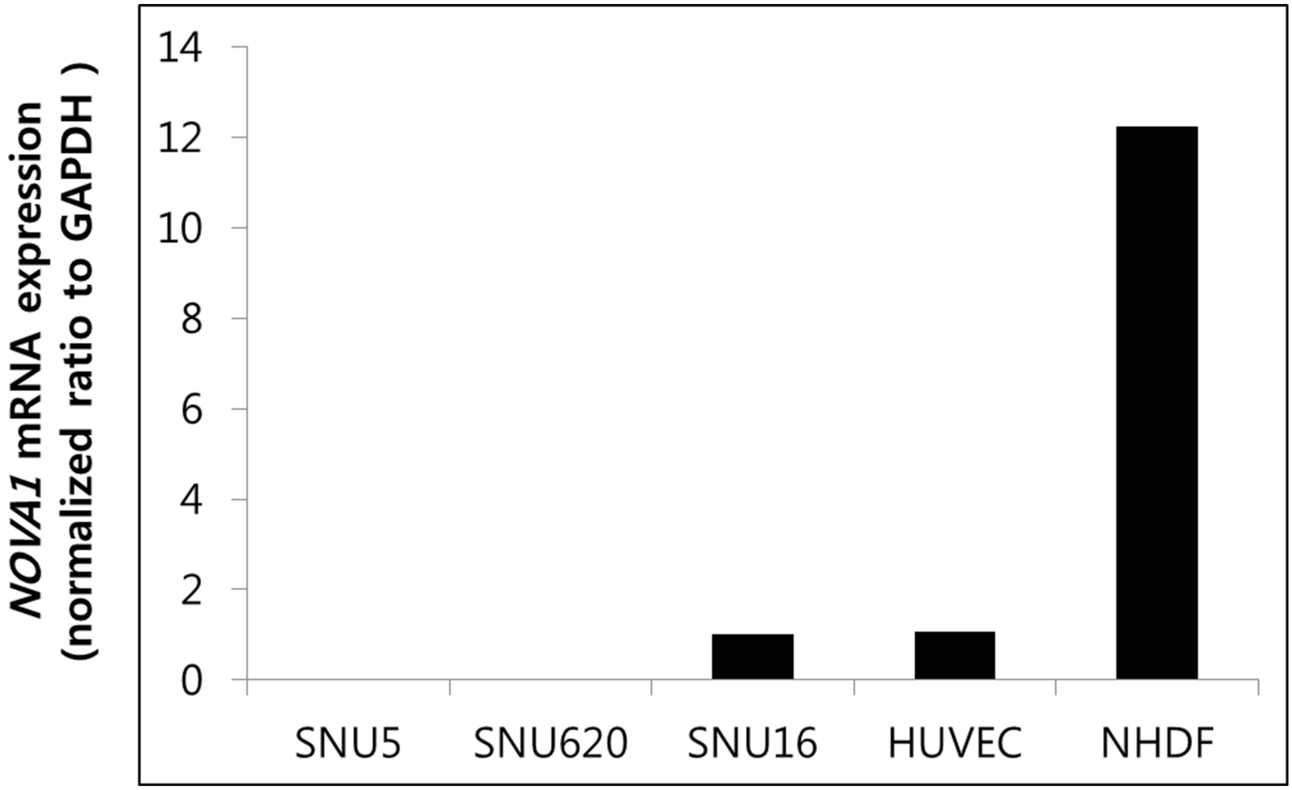

Supplementary Figure S2: Endogenous NOVA1 gene expression in the cell lines of gastric cancer cells, endothelial cells, and fibroblasts. Endogenous NOVA1 gene expression was higher in NHDF cells than the other tested cells; HUVEC and SNU16 cells expressed NOVA1 at low levels while SNU5 or SUN620 cells did not express NOVA1.

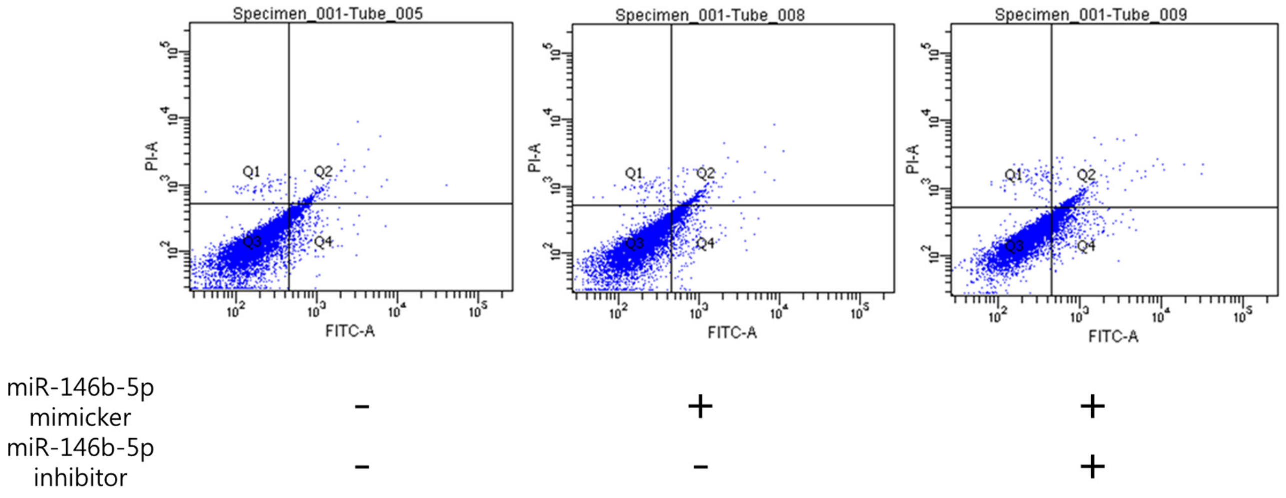

Supplementary Figure S3: Annexin V/ PI assay after transfection of miR-146b-5p. Annexin V/ PI assay showed no significant changes of cell death (apoptosis and necrosis) in NHDF in response to miR-146b-5p manipulation during the 72-hours observation period.

Supplementary Table S1: Characteristics of positive and negative controls

Supplementary Table S2: Correlation of clinico-pathological factors according to miR146b-5p and miR-150–5p expression

Supplementary Table S3: Multivariate cox analysis for overall survival

Supplementary Table S4: Expression of target genes according to miR-146b-5p expression

Supplementary Table S5: Clinico-pathological factors of the set of proximal margin tissues obtained from 140 advanced gastric cancer cases with curative R0 resection

Supplementary Table S6: Sequences of primers

Supplementary Table S7: Details of control samples used in searching target genes

Supplementary Table S8: The characteristics of gastric cancer patients
