# Supplementary material for: NOVA1 inhibition by miR-146b-5p in the remnant tissue microenvironment defines occult residual disease after gastric cancer removal
Source: Oncotarget. 2015 Dec 9;7(3):2475–95. doi: 10.18632/oncotarget.6542 (PMC4823049; doi:10.18632/oncotarget.6542)
Supplement: Supplementary file 3 [file oncotarget-07-2475-s003.docx]

Supplementary Table 1. Characteristics of positive and negative controls

|  | Gastrectomy type | | Macroscopic distance (cm)^a^ | Microscopic distance (cm)^b^ | TNM stage^c^ | Stage^c^ | Histology | Outcome | Survival time (months) | Tumor size (cm) |
| --- | --- | --- | --- | --- | --- | --- | --- | --- | --- | --- |
| Positive control1 | | STG | 2.5 | 0.0 | T3N3M0 | 3C | undifferentiated | Died of cancer recurrence | 20.0 | 5 |
| Positive control2 | | STG | 2.5 | 0.0 | T4N2M1 | 3C | undifferentiated | Died of cancer recurrence | 21.1 | 9 |
| Positive control3 | | STG | 3.0 | 0.0 | T4N0M0 | 2B | undifferentiated | Died of cancer recurrence | 5.5 | 8 |
| Positive control4 | | STG | 4.0 | 0.0 | T4N2M0 | 3C | differentiated | Died of cancer recurrence | 38.6 | 7 |
| Negative control1 | | STG | 5.0 | 5.0 | T3N0M0 | 2B | differentiated | Survived without event | 71.5 | 4 |
| Negative control2 | | STG | 4.0 | 4.0 | T4N2M0 | 3B | undifferentiated | Survived without event | 78.2 | 6 |
| Negative control3 | | STG | 4.0 | 4.0 | T3N1M0 | 2B | undifferentiated | Survived without event | 78.7 | 1 |
| Negative control4 | | STG | 3.5 | 3.5 | T3N1M0 | 3A | differentiated | Survived without event | 80.4 | 1.5 |

^a^ Macroscopic distance of tested area from the original mass edge

^b^ Microscopic distance of tested area from the original mass edge

^c^ The stomach cancers were staged based on the AJCC Cancer Staging Manual, 7th edition.

Supplementary Table 2. Correlation of clinico-pathological factors according to miR146b-5p and miR-150-5p expression

| Characteristic | Patients | | miR146b-5p | | | miR150-5p | | |
| --- | --- | --- | --- | --- | --- | --- | --- | --- |
|  | n | % | Non-high, % | High, % | P value | Non-high, % | High, % | P value |
| All patients | 140 |  |  |  |  |  |  |  |
| Sex |  |  |  |  |  |  |  |  |
| Female | 45 | 32.1 | 41.9 | 24.4 | 0.027 | 67.1 | 69.1 | 0.801 |
| Male | 95 | 67.9 | 58.1 | 75.6 |  | 32.9 | 30.9 |  |
| Age ( years) |  |  |  |  |  |  |  |  |
| Median | 61 |  |  |  |  |  |  |  |
| Range | 25-86 |  |  |  |  |  |  |  |
| Age ( years) |  |  |  |  |  |  |  |  |
| Age ≤60 | 69 | 49.3 | 50.0 | 48.7 | 0.88 | 52.9 | 43.6 | 0.282 |
| Age >60 | 71 | 50.7 | 50.0 | 51.3 |  | 47.1 | 56.4 |  |
| Histologic type |  |  |  |  |  |  |  |  |
| Differentiated | 45 | 32.1 | 32.3 | 32.1 | 0.979 | 36.5 | 25.5 | 0.173 |
| Undifferentiated | 95 | 67.9 | 67.7 | 67.9 |  | 63.5 | 74.5 |  |
| Lymphovascular invasion |  |  |  |  |  |  |  |  |
| Absent | 61 | 50.8 | 53.3 | 48.3 | 0.584 | 54.2 | 45.8 | 0.371 |
| Present | 59 | 49.2 | 46.7 | 51.7 |  | 45.8 | 54.2 |  |
| Method of gastrectomy |  |  |  |  |  |  |  |  |
| Distal gastrectomy | 68 | 48.6 | 54.8 | 43.6 | 0.186 | 47.1 | 50.9 | 0.656 |
| Total gastrectomy | 72 | 51.4 | 45.2 | 56.4 |  | 52.9 | 49.1 |  |
| pT stage |  |  |  |  |  |  |  |  |
| pT2 | 33 | 23.6 | 24.2 | 23.1 | 0.982 | 29.4 | 14.5 | 0.072 |
| pT3 | 54 | 38.6 | 38.7 | 38.5 |  | 38.8 | 38.2 |  |
| pT4 | 53 | 37.9 | 37.1 | 38.5 |  | 31.8 | 47.3 |  |
| pN stage |  |  |  |  |  |  |  |  |
| pN0 | 54 | 38.6 | 46.8 | 32.1 | 0.05 | 47.1 | 25.5 | 0.008* |
| pN1 | 26 | 18.6 | 22.6 | 15.4 |  | 21.2 | 14.5 |  |
| pN2 | 25 | 17.9 | 9.7 | 24.4 |  | 15.3 | 21.8 |  |
| pN3 | 35 | 25 | 21.0 | 28.2 |  | 16.5 | 38.2 |  |
| Intraperitoneal recurrence^a,c^ |  |  |  |  |  |  |  |  |
| Absent | 103 | 87.3 | 94.3 | 81.5 | 0.038* | 91.9 | 79.5 | 0.052 |
| Present | 15 | 12.7 | 5.7 | 18.5 |  | 8.1 | 20.5 |  |
| Intraperitoneal recurrence and/or distant metastasis^b,c^ |  |  |  |  |  |  |  |  |
| Absent | 96 | 81.4 | 90.6 | 73.8 | 0.02* | 86.5 | 72.7 | 0.063 |
| Present | 22 | 18.6 | 9.4 | 26.2 |  | 13.5 | 27.3 |  |

^a^Intraperitoneal recurrence was determined when tumor recurrence occurred in the form of peritoneal carcinomatosis, tumor recurrence at anastomosed sites, or metastasis to intraperitoneal organs/sites/lymph nodes during the follow up observation after surgery and adjuvant therapy.

^b^Intraperitoneal recurrence and/or distant metastasis was determined when intraperitoneal recurrence and/or distant metastasis to extraperitoneal organs such as brain, lung, ovary, or extraperitoneal lymph nodes.

^c^Information not available in some cases due to lack of reliable information for intraperitoneal recurrence and/or distant metastasis. Valid percentage is presented in the table.

Statistically significant differences are indicated by *, which signify two-sided *P* < 0.05, respectively, as determined using the Chi-square test or one-way ANOVA test.

Supplementary Table 3. Multivariate cox analysis for overall survival

| Variables | Category | Univariate analysis | | | Multivariate analysis | | |
| --- | --- | --- | --- | --- | --- | --- | --- |
|  |  | P value | Hazard ratio | 95.0% CI | P value | Hazard ratio | 95.0% CI |
| Sex | Female |  |  |  |  |  |  |
|  | Male | 0.133 | 1.8 | 0.8-3.7 |  |  |  |
| Age ( years) | Age ≤60 |  |  |  |  |  |  |
|  | Age >60 | 0.518 | 1.2 | 0.7-2.3 |  |  |  |
| Histologic type | Differentiated |  |  |  |  |  |  |
|  | Undifferentiated | 0.405 | 1.3 | 0.7-2.7 |  |  |  |
| Lymphovascular invasion | Absent |  |  |  |  |  |  |
|  | Present | 0.019 | 2.5 | 1.2-5.2 | 0.939 | 1.0 | 0.4-2.6 |
| Method of gastrectomy | Distal gastrectomy |  |  |  |  |  |  |
|  | Total gastrectomy | 0.306 | 1.4 | 0.7-2.6 |  |  |  |
| Safety margin from proximal resection margin | >4cm |  |  |  |  |  |  |
|  | >3cm and ≤4cm | 0.019 | 11.2 | 1.5-84.4 | 0.931 | 1.1 | 0.1-10.4 |
|  | >2cm and ≤ 3cm | 0.124 | 5.3 | 0.6-43.8 | 0.554 | 0.5 | 0.0-5.7 |
|  | >1cm and ≤ 2cm | 0.091 | 5.8 | 0.8-44.6 | 0.907 | 0.9 | 0.1-8.2 |
|  | ≤1cm | 0.268 | 3.5 | 0.4-30.9 | 0.649 | 0.6 | 0.0-6.8 |
| Safety margin from distal resection margin | >4cm |  |  |  |  |  |  |
|  | >3cm and ≤4cm | <0.001 | 15.7 | 5.8-42.2 | 0.677 | 1.4 | 0.3-6.3 |
|  | >2cm and ≤ 3cm | 0.068 | 3.9 | 0.9-16.3 | 0.642 | 1.9 | 0.1-30.5 |
|  | >1cm and ≤ 2cm | 0.301 | 1.7 | 0.6-5.0 | 0.715 | 0.7 | 0.1-3.7 |
|  | ≤1cm | 0.9 | 1.1 | 0.4-3.1 | 0.853 | 0.9 | 0.3-3.0 |
| pT stage | pT2 |  |  |  |  |  |  |
|  | pT3 | 0.089 | 6.0 | 0.8-47.3 | 0.342 | 2.9 | 0.3-25.8 |
|  | pT4 | 0.001 | 26.7 | 3.6-195.9 | 0.008 | 17.8 | 2.1-148.2 |
| pN stage | pN0 |  |  |  |  |  |  |
|  | pN1 | 0.168 | 2.2 | 0.7-6.9 | 0.184 | 2.9 | 0.6-13.9 |
|  | pN2 | 0.169 | 2.2 | 0.7-6.9 | 0.466 | 1.8 | 0.4-9.2 |
|  | pN3 | 0 | 8.8 | 3.5-21.7 | 0.019 | 6.4 | 1.4-29.7 |
| miR-146b-5p expression | Non-high |  |  |  |  |  |  |
|  | High | 0.02 | 2.3 | 1.1-4.6 | 0.029 | 3.1 | 1.1-8.6 |
| miR-150-5p expression | Non-high |  |  |  |  |  |  |
|  | High | 0.005 | 2.4 | 1.3-4.6 | 0.772 | 0.9 | 0.4-2.1 |

Supplementary Table 4. Expression of target genes according to miR-146b-5p expression

| Gene | GenBank Accession No. | Correlation | | Mean value of target genes | | |  |
| --- | --- | --- | --- | --- | --- | --- | --- |
|  |  | Coefficient, r | P value | High miR-146b-5p group | Non-high miR-146b-5p group | P value |  |
| BCORL1 | NM_021946.4 | -0.24 | 0.167 | 67.7 | 73.2 | 0.424 |  |
| CCDC117 | NM_173510.2 | 0.26 | 0.128 | 245.7 | 203.4 | 0.154 |  |
| DLGAP1 | NM_001003809.2 | -0.02 | 0.898 | 4.4 | 4.3 | 0.808 |  |
| DTNA | NM_001390.4 | -0.24 | 0.173 | 143.4 | 147.5 | 0.808 |  |
| EGR3 | NM_004430.2 | -0.09 | 0.609 | 151.9 | 187.0 | 0.154 |  |
| FBXL10 | NM_001005366.1 | 0.37 | 0.03* | 220.1 | 167.1 | 0.003* |  |
| HMBOX1 | NM_024567.3 | -0.17 | 0.338 | 346.4 | 332.9 | 0.237 |  |
| HNRNPD | NM_002138.3 | -0.14 | 0.418 | 1576.4 | 1493.0 | 0.281 |  |
| KCTD15 | NM_024076.2 | 0.04 | 0.837 | 256.9 | 223.1 | 0.135 |  |
| LRRC15 | NM_001135057.2 | 0.31 | 0.072 | 20.3 | 17.5 | 0.808 |  |
| MPPE1 | NM_023075.4 | -0.07 | 0.691 | 142.8 | 145.9 | 0.972 |  |
| MXD4 | NM_006454.2 | -0.04 | 0.813 | 98.4 | 96.4 | 0.314 |  |
| MYT1 | NM_004535.2 | 0.02 | 0.915 | 8.9 | 7.1 | 0.366 |  |
| NOVA1 | NM_006491.2 | -0.40 | 0.018* | 50.8 | 65.8 | 0.034* |  |
| NUMB | NM_001005743.1 | -0.31 | 0.067 | 468.4 | 482.7 | 0.917 |  |
| RARB | NM_000965.3 | -0.02 | 0.931 | 48.6 | 42.5 | 0.348 |  |
| RFXDC2 | NM_022841.5 | 0.10 | 0.585 | 182.1 | 156.0 | 0.224 |  |
| RUNX1T1 | NM_004349.2 | -0.14 | 0.433 | 83.6 | 107.8 | 0.082 |  |
| SMAD4 | NM_005359.3 | -0.15 | 0.38 | 520.1 | 538.5 | 0.945 |  |
| SRSF6 | NM_006275.5 | -0.25 | 0.143 | 1237.1 | 1237.2 | 0.509 |  |
| STRBP | NM_001171137.1 | -0.06 | 0.733 | 552.0 | 505.9 | 0.972 |  |
| STX3 | NM_004177.3 | -0.19 | 0.265 | 248.6 | 245.7 | 0.702 |  |
| SYNPR | NM_001130003.1 | 0.61 | <0.001* | 12.8 | 8.1 | 0.728 |  |
| SYT1 | NM_005639.2 | -0.02 | 0.905 | 28.7 | 26.8 | 0.728 |  |
| TRAF6 | NM_145803.1 | 0.18 | 0.29 | 221.9 | 195.3 | 0.020* |  |
| USP3 | NM_006537.2 | 0.02 | 0.892 | 132.6 | 124.0 | 0.602 |  |
| VASN | NM_138440.2 | 0.13 | 0.471 | 55.3 | 56.4 | 0.651 |  |

The expression level (normalized ratio) of each target gene is correlated with the expression level (normalized ratio) of miR-146b-5p. The mean value of normalized ratio of each target gene is compared between high miR-146b-5p expression group and non-high miR-146b-5p expression group. Statistically significant differences are indicated by *, which signify two-sided *P* < 0.05, respectively, as determined using the Pearson correlation test .

Supplementary Table 5. Clinico-pathological factors of the set of proximal margin tissues obtained from 140 advanced gastric cancer cases with curative R0 resection

| Characteristic | Patients | |
| --- | --- | --- |
|  | n | % |
| All patients | 140 |  |
| Sex |  |  |
| Female | 45 | 32.1 |
| Male | 95 | 67.9 |
| Age ( years) |  |  |
| Median | 61 |  |
| Range | 25-86 |  |
| Age ( years) |  |  |
| Age ≤60 | 69 | 49.3 |
| Age >60 | 71 | 50.7 |
| Histologic type |  |  |
| Differentiated | 45 | 32.1 |
| Undifferentiated | 95 | 67.9 |
| Lymphovascular invasion |  |  |
| Absent | 61 | 50.8 |
| Present | 59 | 49.2 |
| Method of gastrectomy |  |  |
| Distal gastrectomy | 68 | 48.6 |
| Total gastrectomy | 72 | 51.4 |
| pT stage |  |  |
| pT2 | 33 | 23.6 |
| pT3 | 54 | 38.6 |
| pT4 | 53 | 37.9 |
| pN stage |  |  |
| pN0 | 54 | 38.6 |
| pN1 | 26 | 18.6 |
| pN2 | 25 | 17.9 |
| pN3 | 35 | 25 |
| Intraperitoneal recurrence^a,c^ |  |  |
| Absent | 103 | 87.3 |
| Present | 15 | 12.7 |
| Intraperitoneal recurrence and/or distant metastasis^b^ |  |  |
| Absent | 96 | 68.6 |
| Present | 44 | 31.4 |

^a^Intraperitoneal recurrence was determined when tumor recurrence occurred in the form of peritoneal carcinomatosis, tumor recurrence at anastomosed sites, or metastasis to intraperitoneal organs/sites/lymph nodes during the follow up observation after surgery and adjuvant therapy.

^b^Intraperitoneal recurrence and/or distant metastasis was determined when intraperitoneal recurrence and/or distant metastasis to extraperitoneal organs such as brain, lung, ovary, or extraperitoneal lymph nodes.

^c^Information not available in some cases due to lack of reliable information for intraperitoneal recurrence. Valid percentage is presented in the table.

Supplementary Table 6. Sequences of primers

| microRNA | mature microRNA | RT primer | PCR primer | |
| --- | --- | --- | --- | --- |
|  |  |  | mir-specific forward primer | universal reverse primer |
| hsa-miR-223-3p | UGUCAGUUUGUCAAAUACCCCA | GTCGTATCCAGTGCAGGGTCCGAGGTATTCGCACTGGATACGACTGGGGT | AAAAAAAAAAGCCCGCTGTCAGTTTGTCAAAT | AAAAAAAAAAGTGCAGGGTCCGAGGT |
| hsa-miR-142-5p | CAUAAAGUAGAAAGCACUACU | GTCGTATCCAGTGCAGGGTCCGAGGTATTCGCACTGGATACGACAGTAGT | AAAAAAAAAAGCCCGCCATAAAGTAGAAAGC | AAAAAAAAAAGTGCAGGGTCCGAGGT |
| hsa-miR-146b-5p | UGAGAACUGAAUUCCAUAGGCU | GTCGTATCCAGTGCAGGGTCCGAGGTATTCGCACTGGATACGACAGCCTA | AAAAAAAAAAGCCCGCTGAGAACTGAATTCCA | AAAAAAAAAAGTGCAGGGTCCGAGGT |
| hsa-miR-150-5p | UCUCCCAACCCUUGUACCAGUG | GTCGTATCCAGTGCAGGGTCCGAGGTATTCGCACTGGATACGACCACTGG | AAAAAAAAAAGCCCGCTCTCCCAACCCTTGTA | AAAAAAAAAAGTGCAGGGTCCGAGGT |
| hsa-miR-362-5p | AAUCCUUGGAACCUAGGUGUGAGU | GTCGTATCCAGTGCAGGGTCCGAGGTATTCGCACTGGATACGACACTCAC | AAAAAAAAAAGCCCGCAATCCTTGGAACCTAG | AAAAAAAAAAGTGCAGGGTCCGAGGT |
| hsa-miR-532-5p | CAUGCCUUGAGUGUAGGACCGU | GTCGTATCCAGTGCAGGGTCCGAGGTATTCGCACTGGATACGACACGGTC | AAAAAAAAAAGCCCGCCATGCCTTGAGTGTAG | AAAAAAAAAAGTGCAGGGTCCGAGGT |
| hsa-miR-502-3p | AAUGCACCUGGGCAAGGAUUCA | GTCGTATCCAGTGCAGGGTCCGAGGTATTCGCACTGGATACGACTGAATC | AAAAAAAAAAGCCCGCAATGCACCTGGGCAAG | AAAAAAAAAAGTGCAGGGTCCGAGGT |
| hsa-miR-1244 | AAGUAGUUGGUUUGUAUGAGAUGGUU | GTCGTATCCAGTGCAGGGTCCGAGGTATTCGCACTGGATACGACAACCAT | AAAAAAAAAAGCCCGCAAGTAGTTGGTTTGTA | AAAAAAAAAAGTGCAGGGTCCGAGGT |
| hsa-miR-132-5p | ACCGUGGCUUUCGAUUGUUACU | GTCGTATCCAGTGCAGGGTCCGAGGTATTCGCACTGGATACGACAGTAAC | AAAAAAAAAAGCCCGCACCGTGGCTTTCGATT | AAAAAAAAAAGTGCAGGGTCCGAGGT |
| hsa-miR-933 | UGUGCGCAGGGAGACCUCUCCC | GTCGTATCCAGTGCAGGGTCCGAGGTATTCGCACTGGATACGACGGGAGA | AAAAAAAAAAGCCCGCTGTGCGCAGGGAGACC | AAAAAAAAAAGTGCAGGGTCCGAGGT |
| hsa-miR-638 | AGGGAUCGCGGGCGGGUGGCGGCCU | GTCGTATCCAGTGCAGGGTCCGAGGTATTCGCACTGGATACGACAGGCCG | AAAAAAAAAAGCCCGCAGGGATCGCGGGCGGG | AAAAAAAAAAGTGCAGGGTCCGAGGT |
| hsa-miR-3195 | CGCGCCGGGCCCGGGUU | GTCGTATCCAGTGCAGGGTCCGAGGTATTCGCACTGGATACGACAACCCG | AAAAAAAAAAGCCCGCCGCGCCGGGCCCGG | AAAAAAAAAAGTGCAGGGTCCGAGGT |
| U6sn | CUGCGCAAGGAUGACACGCAAAUUCGUGAAGCGUUCCAUAUUUUU | GTCGTATCCAGTGCAGGGTCCGAGGTATTCGCACTGGATACGACTCACGA | AAAAAAAAAAGCCCGCCTGCGCAAGGATGAC | AAAAAAAAAAGTGCAGGGTCCGAGGT |

Supplementary Table 7. Details of control samples used in searching target genes

|  |  | Gastrectomy type | Macroscopic distance (cm) | TNM stage | Histology | Outcome | survival time (days) | hsa-miR-146b-5p | hsa-miR-150-5p |
| --- | --- | --- | --- | --- | --- | --- | --- | --- | --- |
| GA-1 | PC-1 | STG | 3.5 | T3N0 | differentiated | died of cancer | 1046 | UP | NO |
| GA-2 | PC-2 | STG | 4 | T3N3 | undifferentiated | died of cancer | 838 | UP | NO |
| GA-3 | PC-3 | STG | 0.5 | T4N3 | differentiated | died of cancer | 613 | UP | UP |
| GA-4 | PC-4 | TG | 4 | T4N3 | undifferentiated | died of cancer | 1394 | UP | UP |
| GA-5 | PC-5 | STG | 0.6 | T3N1 | undifferentiated | died of cancer | 61 | UP | UP |
| GA-6 | PC-6 | TG | 2 | T4N3 | undifferentiated | died of cancer | 2127 | UP | UP |
| GA-7 | PC-7 | TG | 0.7 | T2N0 | undifferentiated | died of cancer | 39 | UP | UP |
| GA-8 | PC-8 | STG | 6 | T4N3 | undifferentiated | died of cancer | 174 | UP | UP |
| GA-9 | PC-9 | TG | 1 | T4N3 | undifferentiated | died of cancer | 120 | UP | UP |
| GA-10 | PC-10 | TG | 3 | T4N0 | undifferentiated | died of cancer | 1086 | UP | UP |
| GA-11 | PC-11 | STG | 7.5 | T4N2 | undifferentiated | died of cancer | 1488 | UP | UP |
| GA-12 | PC-12 | STG | 10 | T2N2 | differentiated | died of cancer | 1045 | UP | UP |
| GB-1 | NC-1 | STG | 3 | T2N1 | differentiated | Survived without event | 2721 | NO | NO |
| GB-2 | NC-2 | TG | 1.5 | T3N1 | undifferentiated | Survived without event | 2734 | NO | NO |
| GB-3 | NC-3 | STG | 1 | T3N2 | undifferentiated | Survived without event | 2585 | NO | NO |
| GB-4 | NC-4 | STG | 3.2 | T4N2 | undifferentiated | Survived without event | 2582 | NO | NO |
| GB-5 | NC-5 | TG | 13.5 | T3N3 | differentiated | Survived without event | 2554 | NO | NO |
| GB-6 | NC-6 | TG | 3 | T4N0 | undifferentiated | Survived without event | 2520 | NO | NO |
| GB-7 | NC-7 | STG | 11 | T3N1 | differentiated | Survived without event | 2473 | NO | NO |
| GB-8 | NC-8 | STG | 5.3 | T3N2 | undifferentiated | Survived without event | 2466 | NO | NO |
| GB-9 | NC-9 | STG | 4 | T4N1 | undifferentiated | Survived without event | 2431 | NO | NO |
| GB-10 | NC-10 | STG | 5 | T4N1 | undifferentiated | Survived without event | 2400 | NO | NO |
| GB-11 | NC-11 | TG | 1.5 | T4N2 | undifferentiated | Survived without event | 2366 | NO | NO |
| GB-12 | NC-12 | STG | 5 | T4N1 | differentiated | Survived without event | 2257 | NO | NO |
| GB-13 | NC-13 | TG | 3.4 | T3N1 | differentiated | Survived without event | 2246 | NO | NO |
| GB-14 | NC-14 | STG | 3 | T2N1 | differentiated | Survived without event | 2046 | NO | NO |
| GB-15 | NC-15 | TG | 4.5 | T3N1 | differentiated | Survived without event | 2163 | NO | NO |
| GB-16 | NC-16 | STG | 5 | T4N0 | undifferentiated | Survived without event | 2140 | NO | NO |
| GB-17 | NC-17 | TG | 3 | T2N2 | undifferentiated | Survived without event | 2102 | NO | NO |
| GB-18 | NC-18 | STG | 5 | T4N2 | undifferentiated | Survived without event | 1907 | NO | NO |
| GB-19 | NC-19 | STG | 9 | T2N1 | differentiated | Survived without event | 2035 | NO | NO |
| GB-20 | NC-20 | STG | 2.5 | T4N0 | undifferentiated | Survived without event | 1775 | NO | NO |
| GB-21 | NC-21 | STG | 2.5 | T3N3 | undifferentiated | Survived without event | 1683 | NO | NO |
| GB-22 | NC-22 | TG | 3 | T2N1 | differentiated | Survived without event | 2541 | NO | NO |
| GB-23 | NC-23 | STG | 4 | T3N1 | differentiated | Survived without event | 2157 | NO | NO |

*Abbreviations: GA, group A; PC, positive control; NC, negative control; STG, subtotal gastrectomy; TG, total gastrectomy

Supplementary Table 8. The characteristics of gastric cancer patients.

| Characteristic | Patients |  |
| --- | --- | --- |
|  | n | % |
| All patients | 250 |  |
| Sex |  |  |
| Male | 172 | 68.8 |
| Female | 78 | 31.2 |
| Age ( years) |  |  |
| Median | 61 |  |
| Range | 29-84 |  |
| Histologic type |  |  |
| Differentiated | 90 | 36 |
| Undifferentiated | 160 | 64 |
| pT stage^a^ |  |  |
| pT2 | 53 | 21.2 |
| pT3 | 58 | 23.2 |
| pT4 | 139 | 55.6 |
| pN stage^a^ |  |  |
| pN0 | 65 | 26 |
| pN1 | 40 | 16 |
| pN2 | 42 | 16.8 |
| pN3 | 103 | 41.2 |
| Distant metastasis^b^ |  |  |
| Absent | 238 | 95.6 |
| Present | 11 | 4.4 |
| Anatomic stage/prognostic group^a^ |  |  |
| I | 28 | 11.2 |
| II | 68 | 27.2 |
| III | 143 | 57.2 |
| IV | 11 | 4.4 |

^a^The stomach cancers were staged based on the AJCC Cancer Staging Manual, 7th edition

^b^ Distatant metastases were determined based on the clinico-pathological information at the time of surgery.
